# Electro‐Organic Synthesis at a Stable Oil/Water/Solid Three‐Phase Interface Reactor

**DOI:** 10.1002/anie.3415969

**Published:** 2026-09-23

**Authors:** Deyin Chen, Jianan Zhu, Huan Wu, Huajun Zheng, Longhao Jiao, Chengpu Chu, Yinghua Xu, Bin Liu

**Affiliations:** ^1^ Zhejiang Key Laboratory of Surface and Interface Science and Engineering for Catalysts State Key Laboratory of Green Chemical Synthesis and Conversion College of Chemical Engineering Zhejiang University of Technology Hangzhou China; ^2^ Department of Materials Science and Engineering City University of Hong Kong Hong Kong SAR China; ^3^ Department of Chemistry Hong Kong Institute for Clean Energy (HKICE) & Center of Super Diamond and Advanced Films (COSDAF) City University of Hong Kong Hong Kong SAR China; ^4^ Institute of Future Technology Zhejiang University of Technology Hangzhou China

**Keywords:** electrochemical reactor, electro‐organic synthesis, oil/water/solid interface, porous membrane electrode, water‐insoluble substrates

## Abstract

Electro‐organic synthesis offers a green and efficient method to produce high‐value organic chemicals under mild conditions, but it suffers from poor water solubility of organic reactant(s) and tedious separation processes for organic product(s) in the conventional electrochemical reaction systems. Herein, we report a porous membrane electrode (PME) reactor design that allows electro‐organic synthesis with concentrated or neat organic reactant(s), achieving product concentrations above 6 M in representative reactions with simplified separation. The PME reactor is demonstrated across several representative electro‐organic transformations, including reduction, oxidation, and oxidation–reduction coupled reactions. The PME reactor can be run at a low operating voltage (∼2 V) with excellent long‐term stability (for more than 400 h) and has achieved the synthesis of pharmaceutical intermediate on a 500‐g scale. The exceptional performance of the PME reactor is associated with its unique interfacial structure, which can create a stable oil/water/solid three‐phase electrochemical reaction interface.

## Graphical abstract

A porous membrane electrode (PME) reactor is developed to promote oil/water/solid three‐phase interfacial contact for electro‐organic synthesis. By promoting the formation of an oil/water interfacial region within or near an amphiphilic catalytic layer, the reactor enables efficient conversion of water‐insoluble organic reactants in aqueous electrolytes with facile product separation and scalable operation.

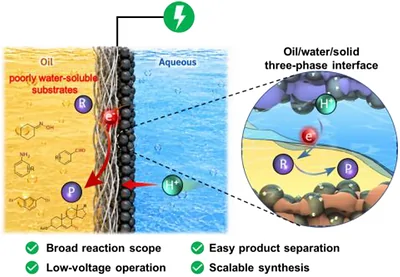

## 1 Introduction

Traditional redox reactions are extensively employed in the industrial synthesis of both bulk and fine organic chemicals, such as the selective hydrogenation of phenylacetylene to styrene, the hydrogenation of nitrobenzene to *p*‐aminophenol, the reductive dechlorination of pentachloropyridine to tetrachloropyridine, the oxidation of *p*‐methoxytoluene to *p*‐methoxybenzaldehyde, and the selective oxidation of cholesterol acetate to 7‐ketocholesterol acetate [1, 2, 3, 4, 5]. These transformations typically rely on chemical reductants or oxidants (e.g., H_2_ [1, 2], Zn powder [3], KMnO_4_ [4], and chromates [4]), which entail explosion hazards and generate secondary pollution. Moreover, many conventional industrial redox processes require harsh conditions such as elevated temperatures and pressures (thermocatalysis), as exemplified by the ammoximation of cyclohexanone to cyclohexanone oxime, [6] the selective hydrogenation of phenylacetylene to styrene [1], and the hydrogenation of nitrobenzene to *p*‐aminophenol [2]. By contrast, electrochemical redox reactions use an applied electrical potential to drive electron‐transfer processes and can potentially be coupled with renewable electricity. Except for paired or redox‐neutral processes, a terminal oxidant or reductant is generally still required; however, electrochemical control can enable the use of milder redox equivalents and provide more tunable and potentially safer reaction conditions. Consequently, electro‐organic synthesis has emerged over the past decade as a vibrant research frontier [7, 8, 9, 10, 11, 12, 13, 14, 15, 16, 17, 18, 19, 20, 21, 22, 23, 24, 25, 26, 27, 28, 29, 30, 31, 32], attracting intense interest from academia and gaining significant traction within industry. Among which, the aqueous‐phase electro‐organic synthesis shows the advantages of low energy consumption and good safety. Unfortunately, the industrial application of aqueous‐phase electro‐organic synthesis is very limited, and only few organic chemicals could be synthesized from high water‐soluble organic reactants [33, 34, 35]. Organic‐phase (hereafter termed the “oil phase”) electro‐organic synthesis addresses the solubility limitations of organic reactants; however, the separation of organic products is more intricate compared to aqueous‐phase electro‐organic synthesis [36, 37, 38, 39]. This is because the electrolyte used in oil‐phase electro‐organic synthesis is costlier, necessitating its separation and purification during the product(s) isolation process.

To overcome the problem of poor organic reactant(s) solubility in aqueous‐phase electro‐organic synthesis, co‐solvent or emulsifier was typically introduced (Figure S1a) [40, 41, 42, 43, 44, 45, 46]. This would not only increase the difficulty for product(s) separation, but also reduce the conductivity of electrolyte, resulting in increased energy consumption. Oil/water/solid three‐phase interfaces have previously been explored to facilitate mass transfer and reactions between immiscible phases [47, 48, 49, 50]. Related multiphase interfacial strategies have also been investigated in organic electrosynthesis [51, 52, 53]. However, their integration into practical electro‐organic reactor architectures remains limited. To further tackle the challenge of product(s) separation in conventional electro‐organic synthesis, Sherbo et al. designed a palladium membrane reactor (Figure S1b), which used palladium membrane to separate the aqueous liquid (electrolyte) from the water‐immiscible organic phase containing the organic solvent and reactant(s). Active hydrogen atoms generated on palladium in the aqueous liquid could selectively pass through the palladium membrane to hydrogenate organic reactant(s) dissolved in the organic solvent in the oil phase [54]. Leitner and coworkers proposed another strategy, in which positively charged palladium‐modified carbon nanotubes acted as a solid emulsifier and electrocatalyst (Figure S1c); the oil and water phase could be automatically separated after the reaction by stopping the ultrasonic disturbance [55]. These two strategies greatly promote electro‐organic synthesis for valuable organic chemicals preparation, unfortunately they are only applicable to the indirect electrochemical reduction reactions with hydrogen atom as the reducing agent.

In this work, a porous membrane electrode (PME) reactor (Figures 1a and S2) is designed and developed, in which the PME is constructed by a layer of hydrophobic conductive material (hydrophobic layer) and a layer of electrocatalyst (catalytic layer). In the PME reactor, the oil phase liquid is placed facing the hydrophobic layer side of the PME, while the aqueous liquid is placed facing the catalytic layer side. The height of aqueous liquid is maintained a bit higher than that of the oil phase liquid. Due to the pressure difference, the oil phase liquid cannot pass through the PME to enter the aqueous liquid. On the other hand, the hydrophobic layer can also block the aqueous liquid from passing through the PME to enter the oil phase liquid. As a result, the oil/water interface is proposed to form within or near the catalytic layer of the PME, providing sites for oil/water/electrocatalyst three‐phase interfaces. Thanks to the good electrical conductivity of the aqueous electrolyte, the electro‐organic synthesis reactions can be operated at low voltages. Furthermore, because the oil phase liquid is in direct contact with the water/electrocatalyst interface, the reactant(s) (pure or dissolved in organic solvent) can quickly transfer to the water/electrocatalyst interface to participate in the electrochemical reaction, and the product(s) can quickly return back to the oil phase after the reaction. Consequently, the PME reactor provides a platform for oil/water/solid triphasic electro‐organic synthesis, addressing both challenges of poor water solubility of organic reactant(s) and tedious separation processes for organic product(s) in conventional electro‐organic synthesis systems.

**FIGURE 1:**
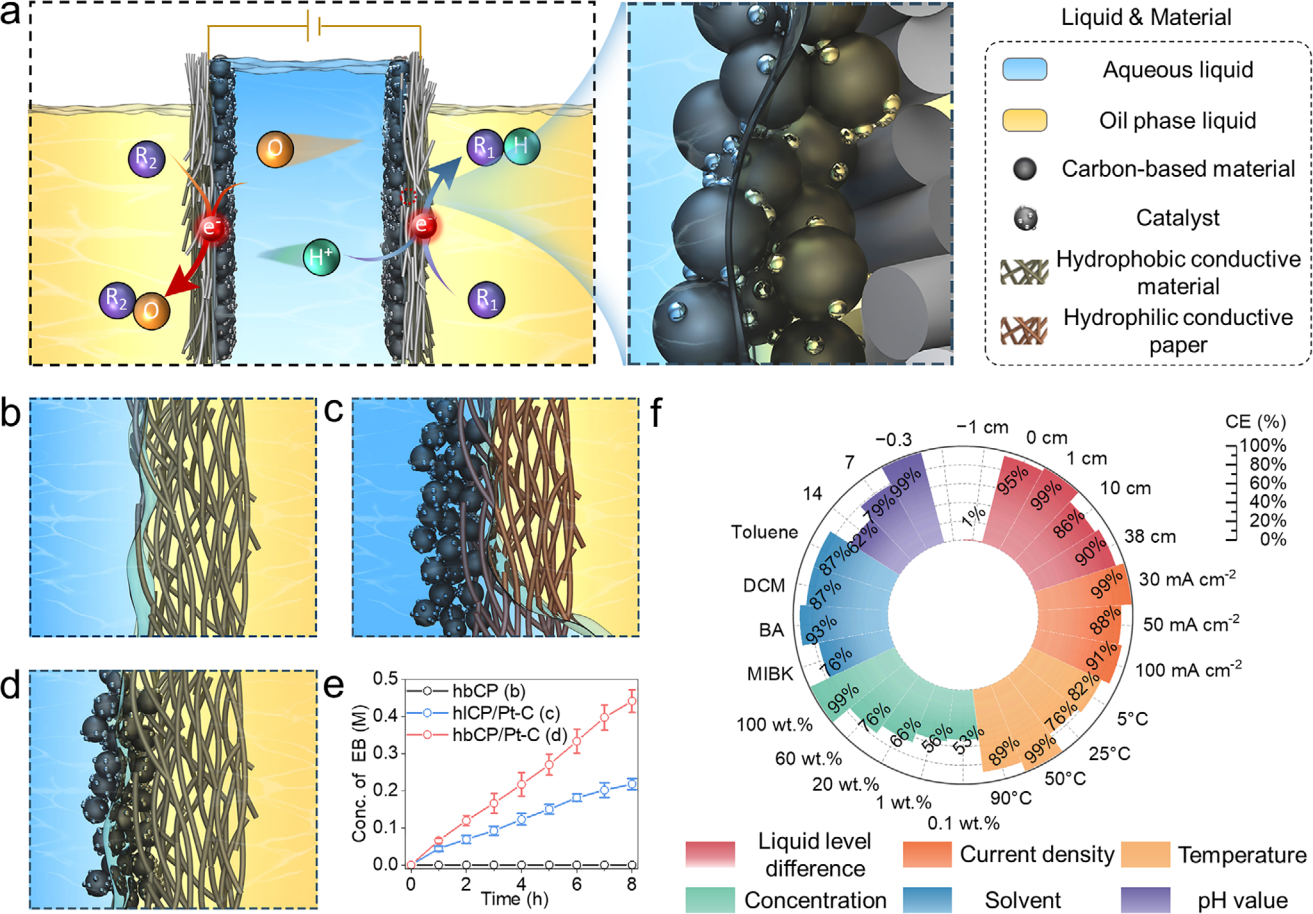
Design and performance optimization of the porous membrane electrode (PME) reactor. (a) Design concept of PME reactor. Styrene hydrogenation conducted in different PME reactors: (b) hbCP PME reactor, (c) hlCP + Pt/C PME reactor, and (d), hbCP + Pt/C PME reactor. (e) Hydrogenation performance of different PME reactors. Conc. represents concentration. (f) Hydrogenation performance of the optimized PME reactor. DCM, BA, and MIBK represent dichloromethane, *n*‐butyl acetate, and methyl isobutyl ketone, respectively. Unless otherwise stated, the styrene hydrogenation reaction was carried out at 50°C and 30 mA cm^−2^. The liquid level difference was 1.0 cm. The chamber diameter is approximately 4.5 cm, with an electrode spacing of 4.0 cm. Detailed reactor dimensions and electrode spacing are provided in Figure S2.

## 2 Results and Discussion

### 2.1 Structure and Operating Parameter Optimization of the PME Reactor

To determine the most effective structure of the PME reactor for electro‐organic synthesis, hydrogenation of styrene was chosen as the model reaction (Figure 1b–e). The results showed that the styrene hydrogenation reaction could hardly occur in the reactor with hydrophobic carbon paper (hbCP; Figure S3a,d) as the PME (Figuree 1b,e). Coating a catalytic layer (Pt/C) on hbCP (hbCP‐Pt/C; Figure S3c,f) greatly increased the styrene hydrogenation efficiency in the PME reactor (Figure 2c,e), but if the hbCP was exchanged by a hydrophilic carbon paper (hlCP) (Figure S3b,e), the styrene hydrogenation efficiency obviously decreased (Figure 2d,e). Thus, the most effective PME composed of a porous hydrophobic conductive layer and a catalytic layer. During electro‐organic synthesis, the hydrophobic layer (hbCP) of the PME was placed facing the oil phase liquid (pure styrene) and the catalytic layer (Pt/C) of the PME was placed facing the aqueous electrolyte (sulfuric acid aqueous solution) in the PME reactor. The level of the sulfuric acid aqueous solution was kept slightly higher than that of the liquid styrene. Due to pressure difference, the styrene and the sulfuric acid aqueous solution were well separated into two compartments by the PME. The oil/water interface is proposed to reside within or near the catalytic layer of the PME (Figures S4 and S5), resulting in the formation of a large number of oil/water/solid three‐phase interfaces. When an electric field was applied, styrene in the oil phase would undergo hydrogenation reaction at the three‐phase interface to produce ethylbenzene (EB). The produced EB then returned back to the oil phase; this would simplify the EB separation process.

**FIGURE 2:**
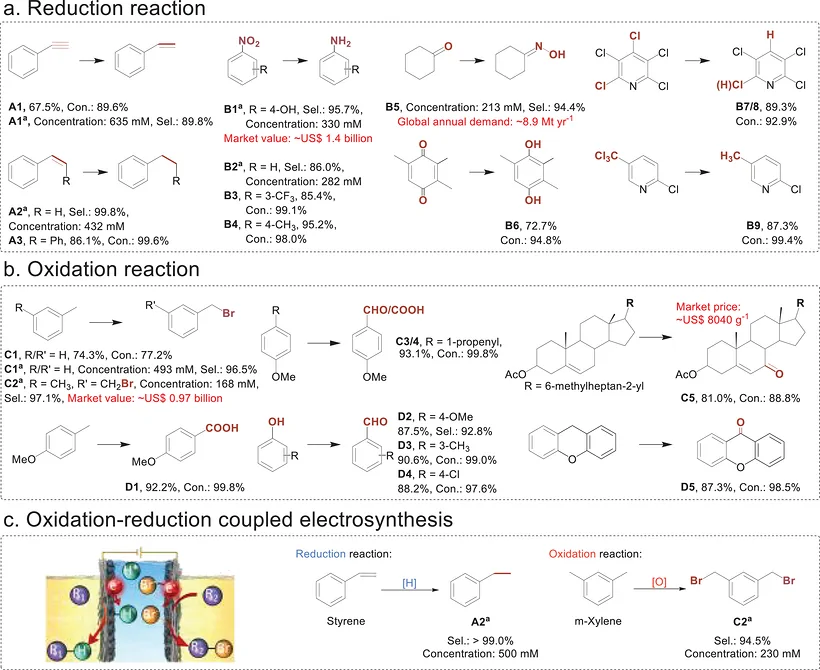
Applicability of the PME reactor to representative electro‐organic transformations. (a) Representative reduction reactions. (b) Representative oxidation reactions. (c) Paired electro‐organic transformations. ^a^Pure reactants were used in the oil phase. Con. represents conversion. Unless otherwise stated, the percentage indicates analytical yields determined by GC or HPLC. A detailed description of the experiment is shown in the section of electrochemical reduction reactions and electrochemical oxidation reactions (Supporting Information and Table S2).

Next, we evaluated the operating parameters of the optimized PME reactor for electro‐organic synthesis (Figure 1f and Table S1). It is found that the PME reactor can achieve high current efficiencies (CEs) under the following reaction conditions: the liquid level difference (between oil phase and aqueous phase) range from 0 to 38 cm, the operating current density range from 30 to 100 mA cm^−2^, the reaction temperature range from 5°C to 90°C, the reactant concentration range from 20 to 100 wt.% (four kinds of organic compounds (ketones, esters, halogenated hydrocarbons, and aromatic hydrocarbons) can be used as the solvent to dissolve organic reactant(s)), and the aqueous electrolyte pH range from 0 to 14. The CEs of the PME reactor could be kept higher than 80% under most conditions, highlighting a very wide operating condition range for the PME reactor.

### 2.2 Applicability of the PME Reactor

Electro‐organic transformations can proceed through predominantly direct electrode–substrate electron transfer or through electrochemically generated reactive intermediates, although these pathways are not necessarily mutually exclusive (Figure S7). To further explore the applicability of the PME configuration beyond the model reaction, several representative reductions, oxidations, and paired electro‐organic transformations were examined (Figures 2).

As shown in Figure 2a, the PME reactor was first evaluated for representative reduction reactions, such as alkene/alkyne selective hydrogenation, which typically relies on precious metal (e.g., platinum, palladium) as the electrocatalyst and generally proceeds via a mediated reduction mechanism [56]. Besides, the PME reactor was also evaluated for other representative electrochemical reduction reactions (Figure 2b). Such reactions are often catalyzed by inexpensive electrocatalysts. It should be emphasized that the synthesis of *p*‐aminophenol (a key intermediate of non‐steroidal anti‐inflammatory drug acetaminophen, 180 000 t yr^−1^, US$1.4 billion) by one‐step electrochemical reduction of nitrobenzene is a widely studied reaction [57, 58]. The PME reactor can not only achieve one‐step electrochemical nitrobenzene reduction to synthesize *p*‐aminophenol without the requirement to add organic cosolvents or emulsifiers, which are commonly used in conventional electro‐organic synthesis systems, but also maintain high reaction selectivity (Figure 2a, B1). Meanwhile, the electrosynthesis of oxime (Figure 2b, B5, a caprolactam intermediate for nylon‐6, 8.9 Mt yr^−1^), vitamin E intermediate D2 (Figure 2b, B6), and polychloropyridines (Figure 2b, B7/8 and B9) for herbicides, further highlights the PME reactor's industrial compatibility [59]. In addition to the electrochemical reduction reactions, the PME reactor was further evaluated for representative electrochemical oxidation reactions (Figures 2b). It is worth pointing out that the PME reactor shows a completely different bromination selectivity as compared to the conventional electrochemical reactor. In the conventional electrochemical reactor, only C(sp^2^)─H can be activated [60], while the PME reactor can activate the relatively inert C(sp^3^)─H to obtain highly selective aromatic side chain bromination products (Figure 2b, C1 and C2). The electrochemical bromination of *m*‐xylene to 1,3‐bis(bromomethyl)benzene (Figure 2b, C2) can be used as a key intermediate step in the alternative to the traditional high‐temperature and high‐pressure synthesis of *m*‐xylylenediamine (polyurethane monomer, US$0.97 billion, Figure S8) [61]. Additionally, the PME reactor can also realize highly selective electrochemical allylic oxidation of cholesterol acetate to 7‐ketocholesterol acetate (Figure 2b, C5), which has important applications in the synthesis of many natural drugs (US$8040 per gram) [62]. Besides, the PME reactor also enables oxidation of aromatic substrates under mild conditions (Figure 2c). Typical examples include the oxidation of 4‐methylanisole (D1) and aromatic alcohols to aldehydes (D2–D4), achieving high selectivity and efficiency without the requirement to add external oxidants. Furthermore, the oxidation of oxanthrene to xanthone (D5, a natural compound with antitumor activity and for fluorescent and fine‐chemical applications) further demonstrates the applicability of the PME reactor to representative oxidation reactions.

Last but not the least, coupled electro‐organic synthesis (reduction + oxidation) can be accomplished in a three‐chamber PME reactor (Figures 2e, S2a, and S9), which can further increase the energy efficiency. Collectively, these representative examples suggest the potential applicability of the PME configuration to reduction, oxidation, and paired electro‐organic transformations. For the three representative reactions examined, the PME consistently operated at lower cell voltages and achieved higher reaction efficiencies and product yields than the corresponding conventional electrochemical configurations (Figure S11).

### 2.3 Practicability of the PME Reactor

To assess the practicability of the PME reactor, a plate‐frame construction (Figures 3a and S10) was adopted. The plate‐frame construction is commonly used for building electrochemical reactors in industry, which can easily expand the production capacity by increasing the electrode area and the number of modules.

**FIGURE 3:**
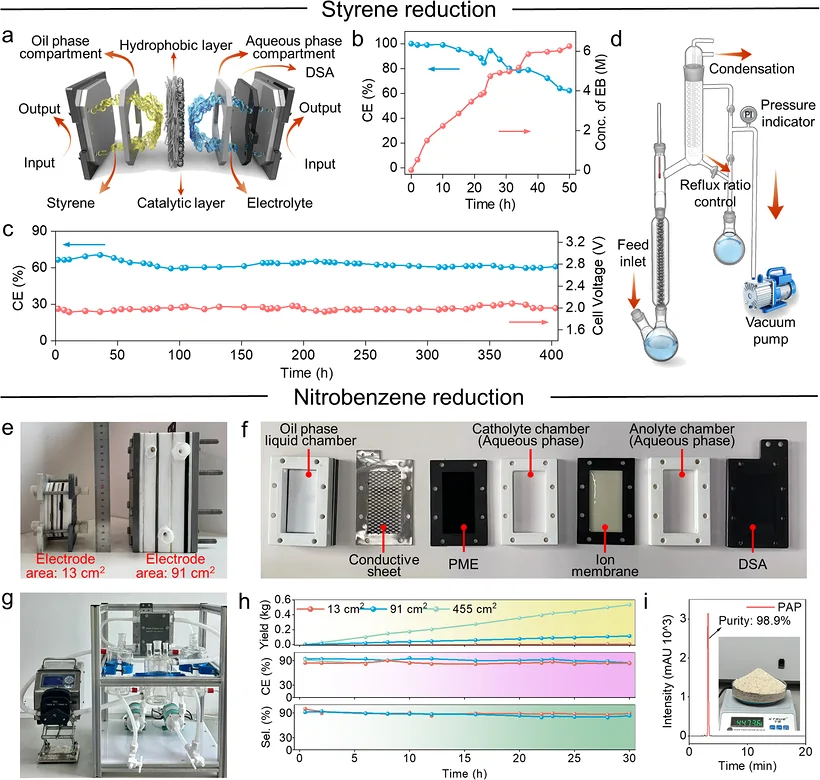
Engineering performance (stability, product concentration, separation, and scale‐up effect) of the PME reactor. (a) A schematic diagram showing the plate‐frame PME reactor. (b) Batch and (c) continuous hydrogenation of pure styrene in the plate‐frame PME reactor operated at the room temperature. (d) A schematic diagram showing the product(s) separation process. (e) Photograph showing the plate‐frame PME reactors with different electrode areas. (f) Exploded view of the large‐sized PME reactor. (g) Photograph showing the electrolysis system equipped with a 91 cm^2^ PME reactor. (h) Comparison of yield, current efficiency (CE) and selectivity (Sel.) for different‐sized PME reactors. The *p*‐aminophenol yield was quantified by HPLC. (i) Mass of purified p‐aminophenol crystal (447.4 g, HPLC purity) obtained after 30 h using a multi‐module PME reactor with a total electrode area of 455 cm^2^.

Figure 3b displays the results of the batch electrolysis experiments using the plate‐frame PME reactor. The product (EB) concentration at the end of the reaction in the oil phase could reach more than 6 M. Such a high product concentration is very difficult to be obtained in conventional electro‐organic synthesis systems due to the low water solubility of organic reactant(s). As shown in Figure 3c, the CE and cell voltage of the plate‐frame PME reactor could be maintained stable in a continuous electrosynthesis mode for more than 400 h. It is worthy to note that the operating voltage of the plate‐frame PME reactor is very low (1.8–2.4 V) in both the batch mode (Figures 3b, S10a, and S12) and the continuous mode (Figures 3c and S10b), much lower than that for the conventional oil‐phase electro‐organic synthesis reactors (usually > 4 V). Additionally, it is worth pointing out that in the continuous plate‐frame PME reactor, the aqueous electrolyte could be used continuously for more than 400 h, and in the styrene‐to‐EB case the high‐purity product (> 98.5%) could be extracted from the oil phase solution by a simple distillation process (Figure S13). Quantitative analysis after 200 h showed limited phase crossover, while the PME remained hydrophobic despite measurable electrolyte and interfacial evolution (Table S3). For conventional aqueous‐phase electro‐organic synthesis with added organic cosolvents or emulsifiers, or oil‐phase electro‐organic synthesis, product(s) separation and electrolyte reuse are very tricky because the reactant(s) and product(s) are miscible with the electrolyte.

We took the electrochemical synthesis of *p*‐aminophenol (PAP) from nitrobenzene, which is urgently needed in the market, as an example, to further demonstrate the practicability of the plate‐frame PME reactor by evaluating the scale‐up effect of electrode area (Figures 3e–i and S14). It was found that when the area of a single PME was 13 cm^2^, the current efficiency and selectivity of the electrochemical reaction could be maintained at about 90%, and the yield of PAP was 17 g after 30 h of reaction. When the area of a single PME was increased to 91 cm^2^ (7 times amplification), the current efficiency and selectivity of the electrochemical reaction could be still kept at approximately 90%, producing 115 g of PAP (6.8 times increase) after 30 h of reaction (Figures 3g,h and S14b). When the electrode area was further enlarged to a total of 455 cm^2^ by combining multiple PME modules (Figure S11a), the reaction could still proceed with satisfactory stability and efficiency (Figure 3h), ultimately producing 447 g of high‐purity PAP (98.9%) after simplified post‐processing (Figures 3i). These results support the good engineering scale‐up potential of the plate‐frame PME reactor.

### 2.4 Operating Mechanism of the PME Reactor

To probe the hypothesis that the oil/water interface of PME is located within the catalytic layer, cryo‐scanning electron microscopy (cryo‐SEM) was employed. Cross‐sectional SEM image and the corresponding elemental mappings of the cryogenically frozen PME sample (Figure 4a,b) prepared using dimethyl siloxane styrene (oil phase) and nickel sulfate aqueous solution (water phase) revealed that the catalytic layer exhibited a compact and dense morphology, while the opposing hydrophobic layer maintained a porous architecture dominated by carbon fiber networks. Thickness measurements of the dense and loose regions in the PME cross‐section corresponded closely to those observed in the pre‐frozen PME structure (Figure S15a), supporting morphological preservation during cryogenic processing. The contents of Pt and Ni elements in the dense region of the cross‐section are significantly higher than those in the loose region (Figure 4b), suggesting that most of the nickel sulfate aqueous solution that penetrates into the PME is mainly located within the catalytic layer. The Si content in the loose region is significantly less than that in the dense region close to the hydrophobic layer, but slightly higher than that in the dense region far from the hydrophobic layer. This result suggests that the oil phase liquid that penetrates into the PME passes through the loose layer (hydrophobic layer) and enters the dense layer (catalytic layer) close to the loose layer.

**FIGURE 4:**
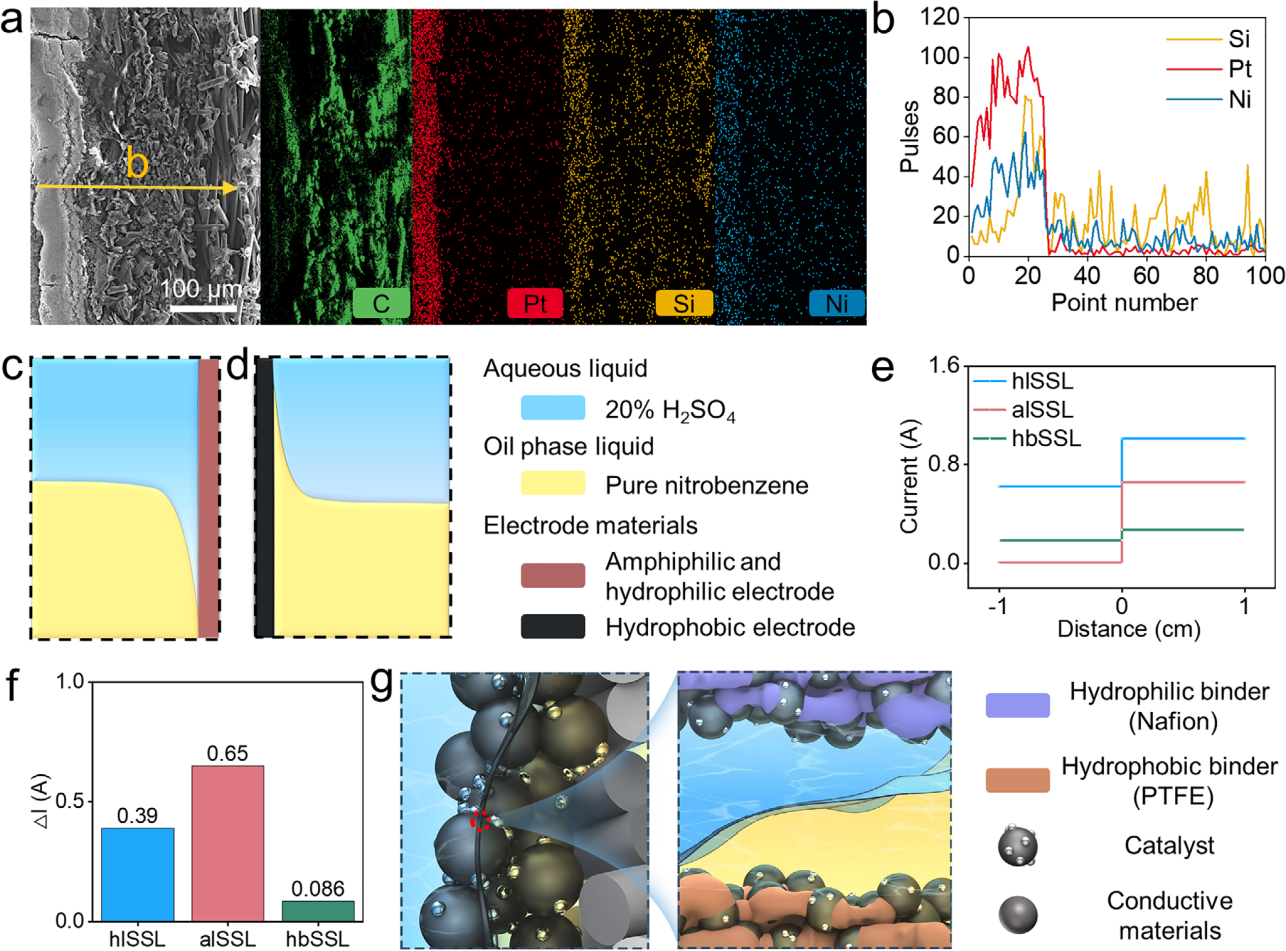
Proposed interfacial mechanism of oil/water/solid three‐phase electrosynthesis in the PME reactor. (a) Cryo‐SEM/EDS analysis and (b) line scan of the PME cross‐section at the oil/water interface after freezing. Schematic illustration of oil/water interface distribution: (c) Hydrophilic or (d) amphiphilic and hydrophobic electrodes in the bisphase system. (e) The current–distance curve measured using hydrophilic, amphiphilic, and hydrophobic electrodes, and (f) the comparison chart of the current step difference. (g) Schematic illustration of oil/water/solid three‐interface in catalytic layer.

Based on these observations, we propose that the primary oil/water interface region is likely located within or near the dense layer (catalytic layer) proximal to the hydrophobic layer boundary. The observed Si depletion in the porous region can be attributed to two synergistic reasons: (a) oil phase contraction during cryo‐solidification (Figure S15b), and (b) weak carbon fiber–oil adhesion, resulting in substantial particulate loss during cryo‐sectioning (Figure S15c).

To clarify the formation and function of the oil/water/solid three‐phase boundary, a simplified model using flat stainless‐steel electrodes was constructed to isolate the interfacial behavior from structural complexity [63]. When immersed in the oil phase, hydrophilic and amphiphilic electrode retained a thin aqueous film on their surface (Figure 4c). In contrast, the hydrophobic electrode was rapidly covered by the oil (Figures 4d and S16d). Chronoamperometric measurements were conducted by sequentially recording current responses in the pure aqueous phase and upon oil phase contact (Figure 4e,f). Electrode with different wettabilities exhibited significantly distinct current steps: amphiphilic electrodes (alSSL, contact angle ∼90°, Figure S16c) exhibited the largest current step (Δ*I* ≈ 0.65 A), indicative of the most favorable diffusion interface (Figure S16); hydrophilic electrodes (hlSSL, contact angle ∼30°, Figure S16b) produced smaller steps (Δ*I* ≈ 0.39 A) possibly due to thicker water films extending the diffusion path; whereas hydrophobic electrodes (hbSSL, contact angle ∼140°, Figures 4d and S16d) were rapidly coated by oil, yielding negligible signals (Δ*I* ≈ 0.006 A). To verify that these findings extend to practical porous configurations, the flat electrode was replaced by carbon paper. The same trend was observed (Figure S17), suggesting that an amphiphilic surface provides the optimal configuration for oil/phase mass transfer. Inspired by existing gas/liquid/solid triphase models [58, 59, 60, 61, 62, 63], we proposed an analogous mechanistic model (Figure 4g): the aqueous phase migrates along Nafion‐modified hydrophilic channels, while the oil phase penetrates the catalyst‐layer pores and preferentially wets PTFE‐modified hydrophobic materials. These two phases are proposed to form continuous thin liquid films across the microporous network, minimizing diffusion distances and generating abundant reactive oil/water/solid triphase interfaces. Under dynamic flow or interfacial perturbation, the oil phase preferentially adheres to amphiphilic conductive surfaces, potentially helping to maintain stable reactive contact. These observations further support partial penetration of the organic phase into the catalytic layer and are consistent with the proposed interfacial model.

To further validate the interfacial model under practical operating conditions, liquid‐level control experiments were performed to examine phase stability. Stable separation and operation were achieved when the aqueous phase was positioned above the oil phase using hbCP (Figure S18a) or hbCP + Pt/C (Figure S18b), whereas using hlCP (Figure S18c) or placing the oil phase above the aqueous phase led to phase interpenetration, voltage increase, and performance loss (Figure S18d). These findings indicate that precise liquid‐level control is critical for maintaining phase separation and are consistent with the proposed interfacial configuration. Direct visualization of the dynamic reactive interface during electrolysis remains challenging, and the present model should therefore be regarded as a mechanistic interpretation supported by the available structural and electrochemical evidence.

### 2.5 Techno‐Economic Analysis (TEA) and Cycle‐Life Assessment (LCA)

Encouraged by the enlarged‐scale performance of the PME reactor, we conducted a preliminary TEA and LCA to benchmark the PME process against conventional industrial routes (Figure 5). Electricity consumption for producing 1 kg of PAP was estimated as E = QU, scaling directly with cell voltage and inversely with current efficiency (Figures S21 and S22; Note S2). Only when the PME reactor operates at a current density higher than 200 mA cm−^2^ does its energy consumption exceed that of conventional electrolysis (36 MJ kg^−1^), whereas under standard conditions (13 MJ kg^−1^) it remains comparable to catalytic hydrogenation (12 MJ kg^−1^) (Figure 5a and Table S8 and TEA spreadsheet). The TEA framework (Figure S22) integrates materials, capital, operating costs, and cost adjustments (Notes S2–S4 and Table S5–S9). Materials cost (67.3%) dominates the main total cost (Figure 5b, US$2113.50 ton^−1^ day^−1^), followed by capital cost (11.9%, consists of electrolyzer, separation equipment and BOP) and electricity cost (8.6%).

**FIGURE 5:**
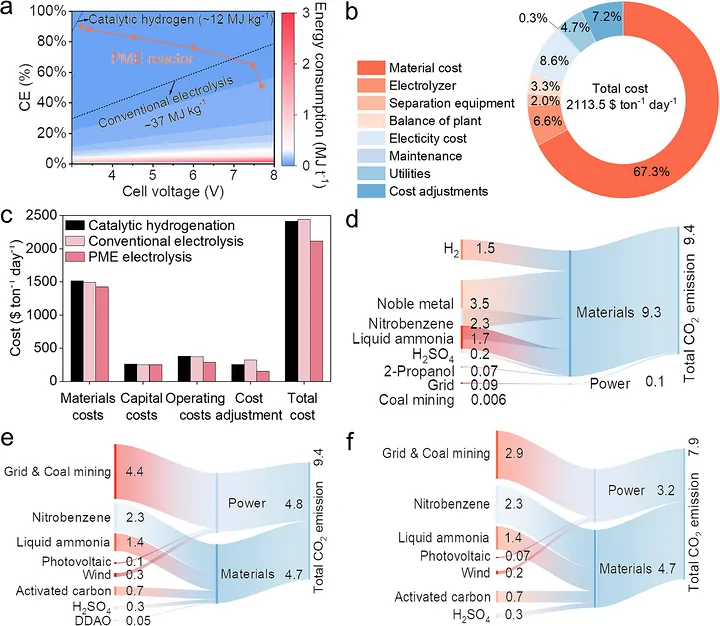
Techno‐economic analysis (TEA) and CO_2_ emission life‐cycle assessment (LCA). (a) Electricity consumption as a function of CE and cell voltage. (b) Total cost for producing one ton of PAP at the condition: the CE of 90%, current density of 50 mA cm−^2^, and cell voltage of 3.23 V. (c) Breakdown of costs for catalytic hydrogenation, conventional electrolysis, and PME electrolysis. Sankey diagram of CO_2_ emission analysis for the (d) catalytic hydrogenation process, (e) conventional electrolysis, and (f) PME electrolysis.

However, the PME reactor reduces overall cost adjustments (Figure 5c) through its simplified downstream process, eliminating the need for high‐pressure H_2_ (2–5 MPa), noble‐metal recovery, emulsifiers or co‐solvents, thereby improving overall process efficiency (Tables S7–S9). At a standard condition (50 mA cm^−1^, 3.23 V, 90% CE), the PME electrolysis route reaches a gross profit of US$486.42 t^−1^ day^−1^ (Note S4). To verify the impact of current density and CE of PAP on profitability, we conducted a sensitivity analysis (Note S5). Sensitivity analysis indicates that profitability is dominated by market price, whereas capital cost decreases sharply with increasing current density (Figures S23a), while operating cost varies modestly with current density and CE (Figure S23b), remaining favorable above ∼2.11 US$ kg^−1^ PAP (Figure S23c). Net present value (NPV) analysis, assuming a 10% capital salvage value, predicts a 100 t day^−1^ PME plant with an end‐of‐life NPV of US$272.6 million and a payback period of 2.8 years, indicating its economic competitiveness under the modeled operating scenario (Figure S23d and Note S6; TEA spreadsheet for details).

Life‐cycle assessment (LCA) reveals that catalytic hydrogenation emits ∼9.4 kgCO_2_e kg^−1^ PAP, largely from noble metal catalysts (∼3.6 kgCO_2_e kg^−1^, Figure 5d and Table S11), while electrolysis methods emit far less CO_2_ than catalytic hydrogenation, with conventional electrolysis emitting ∼9.1 kgCO_2_e kg^−1^ PAP, while PME electrolysis emits only ∼7.6 kgCO_2_e kg^−1^ PAP when using grid electricity and coal mining (Figure 5e,f; Tables S12 and S13). When powered by wind or photovoltaic electricity, CO_2_ emissions of electrolysis methods can further drop to ∼4.8–5.1 kgCO2e kg^−1^ (Figures 5e,f and S25; Tables S12 and S13), achieving ∼48% reduction relative to catalytic hydrogenation process. Additional process‐level sustainability metrics further show that PME electrolysis reduces auxiliary‐material intensity, separation‐energy intensity, acidification potential, and fossil‐resource depletion by 18.2%, 35.0%, 10.8%, and 12.3%, respectively, compared with conventional electrolysis (Table S14). These results provide a preliminary process‐level perspective on the sustainability implications of the three PAP production routes, rather than a comprehensive sustainability assessment (Note S8). Thus, these analyses suggest that PME electrolysis could represent a potentially lower‐carbon and economically competitive alternative for PAP production under the modeled scenarios, while offering potential process and safety advantages and further decarbonization opportunities when powered by renewable electricity.

## 3 Conclusion

In this work, a porous membrane electrode (PME) reactor is developed for electro‐organic synthesis at a stable oil/water/solid three‐phase electrochemical reaction interface. The PME is composed of a porous hydrophobic layer and a catalytic layer. The hydrophobic layer of the PME is placed facing the oil phase liquid, while the catalytic layer of the PME is placed facing the aqueous electrolyte. During PME reactor operation, the level of the aqueous liquid is kept slightly above the oil phase to suppress phase interpenetration, which is consistent with the formation of oil/water/solid interfacial regions within or near the catalytic layer. The PME reactor provides a triphasic reaction platform that is demonstrated across several representative electro‐organic transformations, enabling electro‐organic synthesis using concentrated or neat, poorly water‐soluble organic reactants without adding electrolyte to the organic phase and at very low operating voltages.

## Author Contributions

**Deyin Chen**: conceptualization, methodology, investigation, writing – original draft, writing – review and editing, visualization. **Jianan Zhu**: investigation, validation, software. **Huan Wu**: investigation, validation, software. **Huajun Zheng**: methodology, resources. **Longhao Jiao**: software, investigation, validation. **Chengpu Chu**: investigation, validation, software. **Yinghua Xu**: conceptualization, writing – review and editing, project administration, funding acquisition, resources. **Bin Liu**: conceptualization, project administration, funding acquisition, resources, writing – review and editing.

## Conflicts of Interest

The authors declare no conflicts of interest.

## Supporting information



The authors have cited additional references within the Supporting Information [64, 65, 66, 67, 68, 69, 70, 71, 72, 73, 74, 75, 76, 77, 78, 79, 80, 81, 82, 83, 84]. **Supporting File**: anie74991‐sup‐0001‐SuppMat.docx.

## Data Availability

The data that support the findings of this study are available from the corresponding author upon reasonable request.
