# Supplementary material for: Electro‐Organic Synthesis at a Stable Oil/Water/Solid Three‐Phase Interface Reactor
Source: Angew Chem Int Ed Engl. 2026 Sep 23;65(46):e3415969. doi: 10.1002/anie.3415969 (PMC13749285; doi:10.1002/anie.3415969)
Supplement: Supplementary file 1 [file ANIE-65-e3415969-s001.docx]

**Supporting Information**

**Electro-Organic Synthesis at a Stable Oil/Water/Solid Three-Phase Interface Reactor**

Deyin Chen^1^, Jianan Zhu^1^, Huan Wu^2,3^, Huajun Zheng^1^, Longhao Jiao^1^, Chengpu Chu^1^, Yinghua Xu^1,4,*^ and Bin Liu^2,3,*^

^1^Zhejiang Key Laboratory of Surface and Interface Science and Engineering for Catalysts, State Key Laboratory of Green Chemical Synthesis and Conversion, College of Chemical Engineering, Zhejiang University of Technology, Hangzhou 310014, China

^2^Department of Materials Science and Engineering, City University of Hong Kong, Hong Kong SAR 999077, China

^3^Department of Chemistry, Hong Kong Institute for Clean Energy (HKICE) & Center of Super Diamond and Advanced Films (COSDAF), City University of Hong Kong, Hong Kong SAR 999077, China

^4^Institute of Future Technology, Zhejiang University of Technology, Hangzhou 310015, China

^*^Corresponding author

E-mail: [xuyh@zjut.edu.cn](mailto:xuyh@zjut.edu.cn) (Y. Xu) and [bliu48@cityu.edu.hk](mailto:bliu48@cityu.edu.hk) (B. Liu)

**EXPERIMENTAL SECTION**

**Materials**

All materials and reagents were purchased from commercial sources and used as received unless otherwise noted. Nitrobenzene (99%), p-aminophenol (98.0%), aniline (99.9%), p-toluidine (99.0%), 3-aminobenzotrifluoride (99%+), 4,4’-diaminodiphenyl ether (98%), cyclohexanone (99.5%), 3-methylpyridine (98.0%), 2-chloro-5-chloromethylpyridine (97%), phenylacetylene (97%+), styrene (99.0%), benzyl bromide (99%), 3-methylbenzyl bromide (96%), anethole (98%), m-methyl benzaldehyde (98%+), xanthone (98%+), toluene (99.5%), m-xylene (99.0%), ethyl acetate (AR, 99.5%), chloroform (CHCl_3_, AR, 99.0%), tert-butyl hydroperoxide (t-BuOOH, 70% aqueous solution), sulfuric acid (H_2_SO_4_, AR, 95−98%), sodium hydroxide (NaOH, AR, 96%), sodium nitrite (NaNO_2_, AR, 99%), isopropanol (AR) and ethanol (EtOH, AR) were purchased from Adamas-beta/Titan. N-phenylhydroxylamine (97%) was purchased from Leyan Chemical. 3-Nitrobenzotrifluoride (GC, 96%) was purchased from TCI-Shanghai. Nitrobenzene (98%), p-nitrotoluene (99.0%), cyclohexanol (GC, 99%), caprolactam (99%), trimethylhydroquinone (98%), 2-bromotoluene (99%), cholesterol acetate, 7-keto-cholesterol acetate, tetrachloro-N-hydroxyphthalimide (Cl_4_NHPI), pyridine (AR) and 4-methyl-2-pentanone (AR, 99.0%) were purchased from Macklin reagent. Cyclohexanone oxime (97%), 2,3,5-trimethylcyclohexa-2,5-diene-1,4-dione (97%), 2,3,6-trichloropyridine (96%), 1-bromo-4-(bromomethyl)-2-methylbenzene (98%), 4-methylanisole (98%), ceric sulfate (Ce(SO_4_)_2_, 97%), sodium bromide (NaBr, 98%) and ammonium bromide (NH_4_Br, 98%) were purchased from Leyan Biomedical. Pentachloropyridine (97%), 2,3,5,6-tetrachloropyridine (98%), 2,3,5-trichloropyridine (98%), 2-chloro-5-methylpyridine (98%), 3-methylbenzyl alcohol (97%) and 4-methoxybenzaldehyde (GC, 99%) were purchased from Meryer Chemical. 2-Chloro-5-trichloromethylpyridine (98%), 4-methoxybenzyl alcohol (98%) and 2-chloro-5-dichloromethylpyridine (98%) were purchased from Weifang Lvba Chemical Industry. Ethylbenzene (standard), sodium perchlorate (NaClO_4_, 99%), 4-chlorobenzaldehyde (98%), 4-chlorobenzoic acid (GC, 99%) and copper (II) nitrate (Cu(NO_3_)_2_, AR, 98%) were purchased from Aladdin. 1,2-Diphenylethane (98%), stilbene (98%), 1,3-bis(bromomethyl)benzene (97%), p-anisic acid (98%), 4-chlorobenzyl alcohol (99.54%), xanthene (98%) and sodium acetate (NaOAc, technical grade) were purchased from Bidepharm.

Methanol (HPLC grade) and acetonitrile (HPLC grade) used for HPLC and GC were obtained from Sinopharm Chemical. Deionized water with a resistivity of 18 MΩ cm, obtained from a Millipore Milli-Q system, was used to prepare all reaction solutions.

XC-72R carbon powder, Monel, platinum (II) chlorate hexahydrate (H_2_PtCl_6_▪6H_2_O), hydrobromic acid (HBr, CP, 40.0%) and palladium/carbon (10 wt.% Pd/C) were purchased from Adamas-beta/Titan. Sodium tetrachloropalladate (II) (NaPdCl_4_, 98%) was purchased from Macklin. Copper powder (100–200 nm) was purchased from Yingtai Metal Materials Co., Ltd. Carbon nanotubes (95%, 8–15 nm) and silver powder (99.5%, 60–120 nm) were purchased from Aladdin. Platinum/carbon (10−40 wt.% Pt/C) was purchased from Sinero Tech. Co., Ltd.

Gases (N_2_, 99.999% pure) were supplied by the JinTe Industrial Gases. Nafion (D520, 5 wt.%, DuPont), carbon paper (YLS-30T), used as the primary porous conductive substrate, hydrophilic carbon paper (SGL28AA), and hydrophilic conductive carbon cloth (SCC130L) were purchased from Sinero Tech. Co., Ltd.

The Nafion 117 membrane, Pt sheet electrode (1 × 1 cm^2^) and Ag/AgCl reference electrode were provided by Hangzhou Saiao Technology ([www.hzcell.com](http://www.hzcell.com)). The gas diffusion electrode (thickness of 5−20 μm) was used as working electrode or electrode support.

**Product(s) quantification**

GC analysis was performed on an Agilent 7890A instrument equipped with a flame ionization detector and a capillary column (HP-INNOWAX, 30 m × 0.32 mm internal diameter, 0.5 μm particle size), using high-purity nitrogen (99.999%) as the carrier gas.

HPLC analysis was performed on a Thermo Scientific Dionex ultimate 3000 HPLC instrument. The HPLC system consisted of a symmetry column (250 mm length × 4.6 mm internal diameter, 5 μm particle size), an injection valve with a 20 μl sample loop and an ultraviolet detector. Standard calibration curves were employed to determine the concentration of products. The precision of the mass balance measurements was 100 ± 5% for GC analysis and 100 ± 3% for HPLC analysis.

Silica gel was used for column chromatography. Analytical thin layer chromatography (TLC) was visualized with UV light.

**Preparation of porous membrane electrode**

An ink method was used to prepare the modified porous membrane electrodes. Unless otherwise specified, YLS-30T carbon paper was used as the porous conductive substrate. Commercial PTFE dispersion (60 wt.%) and Nafion solution (20 wt.%) were used as the binder and hydrophilic regulator, respectively, and an isopropanol/ethanol mixture (3:1, v/v; 40 mL) was used as the dispersant. The Nafion/PTFE ratio and catalyst loading were further screened using styrene hydrogenation to establish the PME preparation conditions, with their effects on electrode wettability and reaction performance summarized in Figures S19 and S20, respectively. The catalyst mass was adjusted according to the geometric electrode area and the desired catalyst loading. After ultrasonication for at least 30 min, the entire catalyst ink was sprayed onto the porous substrate on a hot plate to obtain the catalyst-modified porous membrane electrode. The resulting catalyst loading, expressed as the catalyst mass per geometric (mg cm^−2^), is summarized in Table S2. A representative morphology of the catalyst-modified PME is shown in Figure S3c.

**Electro-organic synthesis experiments**

Electro-organic synthesis was conducted in three types of two-chamber (Figures S2b and S10) or three-chamber (Figures 3f and S2a) PME reactors. The electrolyte was added to the aqueous chamber, while the neat organic reactant or its solution in an organic solvent was added to the oil-phase chamber (Figure S2). Unless otherwise stated, batch H-cell experiments were conducted under continuous magnetic stirring. For styrene hydrogenation in the 13 cm^2^ plate-frame PME reactor, the flow rates of 10 wt.% H_2_SO_4_ were 79 and 86 mL min⁻^1^ in the continuous and batch modes (Figures S10a and S10b), respectively, while the corresponding styrene flow rates were 0.38 and 3.75 mL min⁻^1^. The applied current densities were 7.5 and 32 mA cm^−2^, respectively. For p-aminophenol electrosynthesis, the flow rates of 20 wt.% H_2_SO_4_ were 93 and 833 mL min⁻^1^ for the 13 and 91 cm^2^ reactors, respectively, with corresponding nitrobenzene flow rates of 3.75 and 400 mL min⁻^1^ (Figure 3). Each electrolysis experiment was repeated at least 3 times under exactly the same conditions to guarantee reproducibility. Unless otherwise stated, all experiments were performed in an air atmosphere at 25 °C.

For the scale-up electrosynthesis of p-aminophenol, multiple PME modules were combined to provide a total electrode area of 455 cm^2^. The reaction was conducted at 80 °C and 50 mA cm^−2^ for 30 h, corresponding to a total current of 22.75 A and a total charge passed of 682.5 Ah (2.457 × 10^6^ C). The current efficiency and product selectivity are shown in Figure 3h. After reaction and purification, 447.4 g of PAP was obtained with an HPLC purity of 98.9% (Figure 3i). Because pure nitrobenzene was used as the organic phase in substantial excess, residual nitrobenzene was expected after electrolysis and was therefore not used to calculate an overall substrate conversion or conventional isolated yield. Instead, the reaction mixture was analyzed for representative reduction byproducts, including aniline and azo/azoxy species, to further evaluate the product selectivity of the scale-up experiment. The reaction mixture after electrolysis was further analyzed for residual nitrobenzene and representative reduction byproducts, including aniline, azobenzene, and azoxybenzene. The corresponding results are summarized in Table S4.

**Electrochemical experiment preparation**

Hydrophilic, amphiphilic and hydrophobic electrodes (2 × 2.5 cm^2^_,_ stainless steel 316 and carbon paper) were employed as working electrodes (WE), which were prepared by surface pretreatments to achieve contact angles of approximately 30°, 90° and 140°, respectively. Electrochemical measurements were performed in a single-chamber glass cell with a biphasic system consisting of an upper aqueous phase (20 wt% H_2_SO_4_, 40 mL) and a lower organic phase (NB, 40 mL) at 80 ^o^C. A saturated Ag/AgCl electrode served as the reference electrode (RE) and a Pt mesh as the counter electrode (CE), both immersed in the aqueous phase. The working electrode (WE) was vertically lowered stepwise from the aqueous phase until it contacted the nitrobenzene phase. At each position, chronoamperometric measurements were carried out under potentiostatic control at –0.6 V (vs Ag/AgCl). The electrode was first maintained in the aqueous phase for 10 min to obtain a steady-state current, and subsequently brought into contact with the organic phase for another 10 min to capture the current response at the oil/water/solid interface.

The current change at the water–oil interface was defined as:

$$I=I_{oil contact}-I_{aqueous}$$

where $I_{aqueous}$ is the steady state current when the electrode was fully immersed in the aqueous phase (20 wt% H_2_SO_4_), and $I_{oil contact}$ is the current when the electrode first contacted the organic phase (NB). Each measurement was repeated at least three times for statistical significance. The results were further compared among hydrophilic, semi-hydrophilic, and hydrophobic electrodes to evaluate the effect of wettability on three-phase boundary formation and interfacial electron transfer.

The chronoamperometry experiments were conducted in an undivided electrolytic cell using a three-electrode system for electrochemical testing, and the data were collected using a CHI660E electrochemical workstation (Chenhua, Shanghai, China). An Ag/AgCl electrode and a platinum electrode were used as the reference electrode and counter electrode, respectively. The electrode potentials, after iR compensation, were recalibrated to the reversible hydrogen electrode (RHE) potential using the following equation:

$$E_{RHE}=E_{Ag/AgCl}+0.1976+0.0591\times pH$$

**Product(s) identification and quantification**

After electrolysis, the products were diluted with methanol or acetonitrile (Figures 2, B7–B10, C3–C5) and analyzed by a gas chromatography (GC) or a high-performance liquid chromatography (HPLC).

The product yield was calculated based on the following equation:

$$Yield\left（ \% \right）=\frac{mol of the formed product}{mol of the initial reactant}\times100\%$$

The conversion was calculated based on the following equation:

$$Conversion\left（ \% \right）=\frac{mol of the consumed reactant}{mol of the initial reactant}\times100\%$$

The product selectivity was calculated based on the following equation:

$$Selectivity\left（ \% \right）=\frac{C_{product}}{C_{product}+C_{byproduct}}\times100\%$$

where C_product_ and C_byproduct_ refer to the concentration of product and byproduct, respectively.

Current efficiency (CE, %), equivalent here to the product Faradaic efficiency (FE), was calculated using the following equation:

$$CE\left( \% \right)=\frac{n\times v\times F}{I\times t}\times100\%$$

where n is the number of electrons transferred per molecule of product; v is the amount of product (mol) produced during electrolysis; F is the Faraday constant (96500 C • mol ^−1^); I is the applied current (A), and t is the electrolysis time (s).

**Characterizations**

The morphology and elemental distribution of the porous membrane electrode loaded with Pt/C catalyst were examined by (cryogenic) scanning electron microscopy (Cryo-SEM or SEM, HITACHI Regulus 8100) equipped with energy-dispersive X-ray spectroscopy (EDS, Oxford Ultim Max 65, or Bruker Flash 6I60 when equipped with Cryo-SEM). The distribution area and depth of the oil/water phases were imaged and analyzed using a confocal laser scanning microscope (CLSM, Leica STELLARIS 5).

**Contact angle measurements**

The contact angle was measured at room temperature using a contact angle system (JY-82C, Dataphysics, Chengde DingSheng) with 16 μL of deionized water as the probe liquid. Measurements were performed by the sessile drop method, with images recorded every 62 ms. The average contact angle was obtained from three different positions on each sample.

**Electrochemical reduction reactions**

**Unsaturated double/triple bond derivatives (A1**–**A3)**

**Styrene (A1).** Cathode: hbCP/Pt-CNT (60 cm^2^, 0.8 mg cm⁻^2^). 1 M NaOH was added to the aqueous phase chamber (220 mL) and toluene solution of phenylacetylene (25 mM, 80 mL) was added to the oil phase chamber. A constant current of 500 mA was applied at 50 ℃ for 19 hours to obtain styrene (**A1**, 67.5% yield, 89.6% conversion). When 80 mL of pure phenylacetylene was added to the oil phase chamber as reactant, styrene (**A1**, 89.8% selectivity, concentration up to 635 mM) could be obtained after 15 hours of reaction under the above conditions.

**Ethylbenzene (A2).** Cathode: hbCP/Pd-C (3.14 cm^2^, 1 mg cm⁻^2^). 10 wt.% H_2_SO_4_ was added to the aqueous phase chamber (50 mL) and pure styrene (30 mL) was added to the oil phase chamber. A constant current of 90 mA was applied at 50 ℃ for 8 hours to obtain ethylbenzene (**A2**, concentration up to 432 mM, 99.8% selectivity).

**1,2-Diphenylethane (A3).** Cathode: hbCP/Pd-C (60 cm^2^, 0.5 mg cm⁻^2^). 10 wt.% H_2_SO_4_ was added to the aqueous phase chamber (150 mL) and toluene solution of stilbene (25 mM, 80 mL) was added to the oil phase chamber. A constant current of 800 mA was applied at 50 ℃ for 5 hours to obtain 1,2-diphenylethane (**A3**, 86.1% yield, 99.6% conversion).

**Nitrobenzene derivatives (B1**–**B9)**

**p-Aminophenol (B1).** Cathode: hbCP/Cu (3 cm^2^, 1.0 mg cm⁻^2^). 10 wt.% H_2_SO_4_ was added to the aqueous phase chamber (50 mL) and pure nitrobenzene (30 mL) was added to the oil phase chamber. A constant current of 150 mA was applied at 80 ℃ for 10 hours to obtain p-aminophenol (**B1**, 95.7% selectivity, concentration up to 330 mM).

**Aniline (B2).** Cathode: hbCP/Pt-C (3 cm^2^, 1.0 mg cm⁻^2^), 5 wt.% H_2_SO_4_ was added to the cathode aqueous phase chamber (50 mL) and 10 wt.% H_2_SO_4_ was added to the anode aqueous phase chamber (50 mL). The two aqueous chambers were separated by a cation exchange membrane (Nafion 117). Pure nitrobenzene (30 mL) was added to the oil phase chamber. A constant current of 150 mA was applied at 50 ℃ for 12 hours to obtain aniline (**B2**, 86.0% selectivity, concentration up to 282 mM).

**3-(Trifluoromethyl)aniline (B3).** Cathode: hbCP/Pt-C (60 cm^2^, 0.5 mg cm⁻^2^). 1 M NaOH was added to the aqueous phase chamber (150 mL) and the solution of 3-nitrobenzotrifluoride (25 mM in toluene, 80 mL) was added to the oil phase chamber. A constant current of 400 mA was applied at 50 ℃ for 6 hours to obtain 3-(trifluoromethyl)aniline (**B3**, 85.4% yield, 99.1% conversion).

**p-Toluidine (B4).** Cathode: hbCP/Cu (60 cm^2^, 1.0 mg cm⁻^2^). 10 wt.% H_2_SO_4_ was added to the aqueous phase chamber (350 mL) and the solution of p-nitrotoluene (25 mM in toluene, 80 mL) was added to the oil phase chamber. A constant current of 1.8 A was applied at 60 ℃ for 9 hours to obtain p-toluidine (**B4**, 95.2% yield, 98.0% conversion).

**Cyclohexanone oxime (B5)**. Cathode: hbCP/Cu-S (60 cm^2^, 0.5 mg cm⁻^2^) [12]. 0.5 M Phosphate buffer solution (PBS) with 1.5 mol NaNO_2_ was used as the catholyte (360 mL) and 10 wt.% H_2_SO_4_ was used as the anolyte (480 mL), and pure cyclohexanone (80 mL) was added to the oil phase chamber. A constant current of 650 mA was applied at room temperature for 10 hours to obtain cyclohexanone oxime (**B5,** 94.4% selectivity, concentration up to 213 mM).

**Trimethylhydroquinone (B6).** Cathode: hbCP/Pd-C (60 cm^2^, 0.5 mg cm⁻^2^). 10 wt.% H_2_SO_4_ was used as the catholyte (360 mL) and the anolyte (480 mL), and toluene solution of 2,3,5-trimethylcyclohexa-2,5-diene-1,4-dione (25 mM, 80 mL) was added to the oil phase chamber. A constant current of 600 mA was applied at 40 ℃ for 10 hours to obtain trimethylhydroquinone (**B6**, 72.65% yield, 94.8% conversion).

**2,3,5,6-Tetrachloropyridine (B7).** Cathode: hbCP/Ag (60 cm^2^, 0.5 mg cm⁻^2^). 1 M NaOH was added to the aqueous phase chamber (150 mL) and toluene solution of pentachloropyridine (25 mM, 80 mL) was added to the oil phase chamber. A constant current of 600 mA was applied at 50 ℃ for 6 hours to obtain 2,3,5,6-tetrachloropyridine (**B7**, 82.7% yield, 92.9% conversion) and 2,3,5-trichloropyridine (**B8**, 6.6% yield).

**2-Chloro-5-methylpyridine (B9).** Cathode: hbCP/Ag (60 cm^2^, 0.5 mg cm⁻^2^). 10 wt.% LiOAc was added to the aqueous phase chamber (150 mL), and toluene solution of 2-chloro-5-trichloromethylpyridine (25 mM, 80 mL) was added to the oil phase chamber. A constant current of 1.8 A was applied at 30 ℃ for 8 hours to obtain 2-chloro-5-methylpyridine (**B9**, 87.3% yield, 99.4% conversion).

**Dichloromethane (B10).** Cathode: hbCP/Cu (3.14 cm^2^, 1 mg cm⁻^2^). PBS solution (30 mL) was added to the aqueous-phase chamber, and chloroform (30 mL) was added to the oil-phase chamber. A constant current density of 50 mA cm⁻^2^ was applied at room temperature for 11 hours to obtain dichloromethane (**B10**, concentration up to 704.91 mM, 71.0% CE).

**Electrochemical oxidation reactions**

**Aromatic hydrocarbon derivatives (C1**–**C5)**

**Benzyl bromide (C1).** Anode: hbCP/Pt-C (3.14 cm^2^, 0.5 mg cm⁻^2^). 40 wt.% NaBr aqueous solution (45 mL) plus 40 wt.% HBr aqueous solution (5 mL) was added to the anode aqueous phase chamber (50 mL), 10 wt.% H_2_SO_4_ was added to the cathode aqueous phase chamber (50 mL) and CHCl_3_ solution of toluene (0.2 M, 80 mL) was added to the oil phase chamber. A constant current of 120 mA was applied at 15 ℃ for 9 hours to obtain benzyl bromide (**C1**, 74.3% yield, 77.2% conversion). When pure toluene (30 mL) was added to the oil phase chamber as reactant, benzyl bromide (**C1**, 493 mM, 96.5% selectivity) could be obtained after 10 hours of reaction under the above conditions.

**1,3-Bis(bromomethyl)benzene (C2).** Anode: hbCP/Pt-C (3.14 cm^2^, 0.5 mg cm^−2^). 40 wt.% NaBr aqueous solution (45 mL) plus 40 wt.% HBr aqueous solution (5 mL) was added to the anode aqueous phase chamber (50 mL), 10 wt.% H_2_SO_4_ was added to the cathode aqueous phase chamber (50 mL) and 30 mL m-xylene was added to the oil phase chamber. A constant current of 150 mA was applied at 45 ℃ for 10 hours to obtain 1,3-bis(bromomethyl)benzene (**C2**, 97.1% selectivity, concentration up to 168 mM).

**Anisaldehyde derivatives (C3**/**C4)**

**4-Methoxybenzaldehyde (C3) and p-Anisic acid (C4).** Anode: hbCP/Pt-C (60 cm^2^, 0.5 mg cm⁻^2^). 4 mmol Ce(SO_4_)_2_ in 10 wt.% H_2_SO_4_ was added to the aqueous phase chamber (160 mL) and the toluene solution of anethole (25 mM) was added to the oil phase chamber. A constant current of 400 mA was applied at 35 ℃ for 6 hours to obtain mixed 4-methoxybenzaldehyde and anisic acid (**C3/C4**, 93.1% yield, 99.8% conversion).

**Steroid derivative**

**7-Ketocholesterol acetate (C5).** Anode: hbCP (60 cm^2^). 0.1 M NaClO_4_ was added to the aqueous phase chamber (160 mL) and a mixed solution of Cl_4_NHPI (0.2 equiv.), pyridine (2 equiv.), t-BuOOH (1.5 equiv.), and cholesterol acetate (25 mM in MIBK) was added to the oil phase chamber. 10 mA mmol⁻^1^ of reactant was applied at 30 ℃ for 6 hours to obtain 7-ketocholesterol acetate (**C5**, 81.0% yield, 88.8% conversion).

**Aromatic hydrocarbon derivatives (D1**–**D5)**

**p-Anisic acid (D1).** Anode: hbCP (60 cm^2^). 10 wt.% H_2_SO_4_ was added to the aqueous phase chamber (160 mL) and toluene solution of 4-methylanisole (25 mM) was added to the oil phase chamber. A constant current of 400 mA was applied at 45 ℃ for 8 hours to obtain mixed p-anisic acid (**D1**, 92.2% yield, 99.8% conversion).

**4-Methoxybenzaldehyde (D2).** Anode: hbCP/β-Ni(OH)_2_ (3.14 cm^2^, 2 mg cm⁻^2^) [23] 0.15 M H_2_SO_4_ was added to the aqueous phase chamber (50 mL) and n-butyl acetate solution of 4-methoxybenzyl alcohol (66.7 mM) was added to the oil phase chamber. A constant current of 30 mA was applied at 50 ℃ for 10 hours to obtain mixed 4-methoxybenzaldehyde (**D2**, 87.5% yield, 92.8% selectivity).

**3-Methylbenzaldehyde (D3).** Anode: hbCP/β-Ni(OH)_2_ (60 cm^2^, 2 mg cm⁻^2^) [23]. 3 M KOH was added to the aqueous phase chamber (160 mL) and n-butyl acetate solution of 4-methylbenzyl alcohol (25 mM) was added to the oil phase chamber. A constant current of 180 mA was applied at 60 ℃ for 8 hours to obtain 4-methylbenzaldehyde (**D3**, 90.6% yield, 99.0% conversion).

**4-Chlorobenzaldehyde (D4).** Anode: hbCP/β-Ni(OH)_2_ (60 cm^2^, 2 mg cm⁻^2^) [23]. 3 M KOH was added to the aqueous phase chamber (160 mL) and methyl isobutyl ketone solution of 4-chlorobenzyl alcohol (25 mM) was added to the oil phase chamber. A constant current of 180 mA was applied at 60 ℃ for 8 hours to obtain mixed 4-methoxybenzaldehyde and anisic acid (**D4**, 88.2% yield, 97.6% conversion).

**Polycyclic Aromatic Hydrocarbons derivatives**

**9H-Xanthen-9-one (D5).** Anode: hbCP (60 cm^2^). 0.15 M H_2_SO_4_ was added to the aqueous phase chamber (160 mL) and n-butyl acetate solution of xanthene (25 mM) was added to the oil phase chamber. A constant current of 120 mA was applied at 60 ℃ for 8 hours to obtain 9H-xanthen-9-one (**D5**, 87.3% yield, 98.5% conversion).

**Figure S1. Schematic illustration of (a) Conventional electrochemical reactor, (b) Palladium membrane electrochemical reactor, and (c) Pd/CNT(+) modified electrochemical reactor.**

**Figure S2. Photographs of the porous membrane electrode (PME) reactor.** (a) Three-chamber configuration consisting of one aqueous-phase chamber and two oil-phase chambers for coupled electro-organic synthesis. (b) Two-chamber configuration for single-side electro-organic synthesis. The diameter of each chamber was approximately 4.5 cm. The distance between the two PME electrodes in the three-chamber reactor was approximately 9.0 cm, while the distance between the PME and counter electrode in the two-chamber reactor was approximately 4.0 cm. The effect of electrode spacing in the two-chamber PME reactor was further evaluated in Figure S6.

**Figure S3. SEM images of (a) hbCP, (b)** **hlCP/Pt-C and (c) hbCP/Pt-C on the side facing the aqueous electrolyte. SEM images of (d) hbCP, (e) hlCP/Pt-C and (f) hbCP/Pt-C on the side facing the oil liquid.** The insert in the upper right corner shows the contact angle of water droplets.

**Figure S4. Cryo-SEM/EDS analysis of the PME cross-section at the oil**–**water interface after freezing.** (a) Cryo-SEM image, (b) line scan and (c) elemental mapping. The cryogenically frozen PME sample was prepared using dimethyl siloxane styrene and nickel sulfate aqueous solution as oil phase and water phase, respectively.

**Figure S5. Confocal laser scanning microscopy (CLSM) characterization experiment.** Confocal 3D reconstruction images of hbCP/Pt-C after impregnation with ICG in aqueous phase (a) only and BODIPY in oil phase (b) only.

In PME reactor, indocyanine green (ICG, 1 mg mL⁻^1^, 50 mL) was added only to the aqueous solution on the catalytic layer side (a), while boron dipyrromethene (BODIPY, 1 mg mL⁻^1^, 30 mL) was added to the styrene solution on the hydrophobic layer side (b), with a liquid level difference of 1.0 cm. After a 2-hour impregnation period and subsequent drying, the PME was placed catalyst-side-up on a glass slide. Three-dimensional interfacial reconstruction was performed using CLSM to simulate the distribution of the oil/water/solid three-phase interface (Fig. S5).

The aqueous phase (fluorescent red), around 50 μm thick, mainly concentrates 250–300 μm from the glass slide, matching the catalytic layer's thickness approximately (Fig.S5a). The wavy distribution at the bottom indicates that the oil–water interface exists in the form of disturbance within the catalytic layer. Meanwhile, the fluorescent green oil phase lies 100–220 μm from the slide and partially overlaps with the aqueous phase (Fig.S5b). These observations suggest partial organic-phase penetration into the catalytic layer, consistent with an oil/water/solid interfacial region within or near the catalytic layer. Due to large pores in the hydrophobic layer, fluorescent dyes scarcely aggregate or get induced, so the fluorescence mostly concentrates near the catalytic-layer-adjacent hydrophobic (dense) layer and a bit of the catalytic layer.

**Figure S6**. **Effect of electrode spacing on styrene hydrogenation in the two-chamber PME reactor.** EB concentration and cell voltage were measured at electrode spacings of 2.0, 4.0, and 6.0 cm using the hbCP/Pt-C electrode. Unless otherwise stated, the reaction was carried out at 50 °C and 30 mA cm⁻^2^ under otherwise identical conditions.

**Figure S7**. **Representative reaction pathways relevant to the electro-organic transformations investigated in the PME reactor.**

**Note:** Representative pathways involving direct electrode–substrate electron transfer, surface-bound reactive intermediates, and solution-phase electrochemically generated species are schematically illustrated. These pathways may coexist and are provided for mechanistic context rather than as mutually exclusive classifications of the reactions shown in Figure 2.

**Figure S8. Comparison of industrial synthesis route and new electrochemical synthesis route of m-xylylenediamine.**

m-Xylylenediamine (MXDA) is an extremely crucial chemical intermediate. Apart from being widely employed in the production of epoxy resin, curing agent, nylon products, coatings, and other series of chemicals, it is also an extremely vital intermediate for polyurethane monomer. The traditional synthesis method of MXDA usually uses m-xylene as the raw material, and successively produces m-xylylenediamine through ammoniation and hydrogenation processes. This reaction has to be carried out under harsh conditions, such as high temperature and in the presence of noble metal catalysts.

The route of electrochemical bromination of m-xylene to synthesize 1,3-bis(bromomethyl)benzene (1,3-BBMB) has many advantages. Compared with the traditional chemical synthesis method, the electrochemical route can synthesize compounds with higher selectivity and yield while avoiding the use of very toxic chemicals, reducing the generation of by-products, enhancing resource utilization, and lowering production costs.

**Figure S9. Coupled electro-organic synthesis.**

Reaction conditions: hbCP/Pt-C (3.14 cm^2^, 1 mg cm⁻^2^) was applied as both the cathode and anode. A mixed solution containing 10 wt.% H_2_SO_4_ aqueous solution and 40 wt.% NaBr (45 mL) + HBr (5 mL) was added to the aqueous phase chamber. Pure styrene (30 mL) and m-xylene (30 mL) were added to the cathodic and anodic oil phase chamber, respectively. The reaction was carried out at 45 °C and 48 mA cm⁻^2^ for 10 hours.

At the end of the reaction, 230 mM of 1,3-BBMB and 500 mM of ethylbenzene (EB) were obtained in the left and right oil-phase liquid.

**Figure S10.** **Photographs showing the plate-frame PME reactor**. (a) Batch and (b) continuous plate-frame PME reactor. (c) Exploded view. (d) Front view. (e) Side view. (f) Top view. (g) Oblique view.

**Figure S11. Reactor-level comparison of representative electro-organic reactions using PME and conventional electrochemical configurations.** (a, b) Schematic illustrations of the PME and conventional electrochemical configurations used for the reactor-level comparisons. (c, d) Styrene hydrogenation, (e, f) p-aminophenol (PAP) electrosynthesis, and (g, h) stilbene hydrogenation in the PME and conventional reactors, respectively. The cell-voltage values shown in panels c–h represent the average values recorded during electrolysis.

Styrene hydrogenation (c,d): Two-chamber reactors; 50 °C, 30 mA cm^−2^, hbCP/Pt-C (3.14 cm^2^, Pt/C loading: 1.0 mg cm^−2^) and Pt anode (1 cm^2^). (1) PME reactor: pure styrene (30 mL) and 10 wt.% H_2_SO_4_ aqueous solution (50 mL) were placed in the oil-phase and aqueous chambers, respectively. Conventional reactor: the catholyte contained 1.0 M styrene, 10 wt.% H_2_SO_4_, and 30 vol.% methanol (50 mL) and was continuously stirred during 8 h electrolysis.

PAP electrosynthesis (e, f): Three-chamber reactors; 80 °C, 50 mA cm^−2^, hbCP (3.14 cm^2^) and Pt anode (1 cm^2^). (1) PME reactor: pure NB (30 mL) and 20 wt.% H_2_SO_4_ aqueous solution (50 mL) were used as the organic phase and catholyte, respectively. (2) Conventional reactor: the catholyte contained 20 wt.% H_2_SO_4_, 6.5 wt.% nitrobenzene, and 0.12 wt.% DDAO (50 mL) and was continuously stirred during 9 h electrolysis.

Stilbene hydrogenation (g, h): Two-chamber reactors; 50 °C, 30 mA cm^−2^, hbCP/Pd-C (3.14 cm^2^, Pd/C loading: 2.5 mg cm^−2^) and Pt anode (1 cm^2^). (1) PME reactor: 25 mM stilbene in toluene was used as the organic phase and 10 wt.% H_2_SO_4_ aqueous solution as the aqueous phase. (2) Conventional reactor: the catholyte contained 25 mM stilbene, 10 wt.% H_2_SO_4_, and 30 vol.% acetonitrile as catholyte (50 mL) and was continuously stirred during 8 h electrolysis.

**Figure S12.** **Performance of styrene hydrogenation reaction.** Voltage and concentration over time in plate-frame PME Reactor (batch mode).

**Figure S13.** **Separation and purification of** **EB in the mixed oil phase.** (a) Photograph showing the distillation setup. (b) Photograph showing the EB receiver bottle (local enlarged image). (c) Engineering drawing of a distillation unit. (d) The purity of obtained EB determined by GC.

**Figure S14. Photographs showing the plate-frame PME scale-up.** (a) Electrolysis system equipped with five 91 cm^2^ PME modules with a total electrode area of 455 cm^2^ PME reactor. (b) Mass of purified p-aminophenol crystal (85.5 g) synthesized from 91 cm^2^ PME reactor after 30 hours of reaction. (c) The purity of obtained PAP determined by HPLC.

**Figure S15. Effect of cryogenic immobilization on PME.** (a) SEM-EDS image and line scan of the PME cross-section. (b) Comparative cryogenic immobilization photos of the oil-water phases before and after liquid nitrogen freezing. (c) Interfacial detachment image of the oil phase post-cryofixation.

**Figure S16. Photographs of electrodes with different wettability in water and oil.** (a) Electrode fully immersed in the aqueous phase. (b) Hydrophilic, (b) amphiphilic, and (c) hydrophobic electrodes in contact with oil phase.

**Figure S17. Effect of porous electrode wettability on oil/water/solid interface behavior and chronoamperometric response.** Photographs and schematic models of (a) hydrophilic (hlCP), (b) amphiphilic (alCP), and (c) hydrophobic (hbCP) electrodes in contact with the oil phase. (d) Chronoamperometric measurements of the three electrodes and comparison of their current step differences (ΔI).

**Figure S18. Photographs showing liquid penetration across the PME.** (a) hbCP, (b) hbCP/Pt-C, (c) hlCP/Pt-C and (d) hbCP/Pt-C.

**Figure S19. Effect of Nafion/PTFE binder composition on the wettability and styrene hydrogenation performance of the PME.** Styrene hydrogenation using hbCP/Pt-C electrodes prepared with (a) 2V Nafion, (b) 1.5V/0.5V Nafion/PTFE, (c) 1V/1V Nafion/PTFE, and (d) 2V PTFE. EB concentration and current efficiency (CE) were monitored during electrolysis, and the inset images show the water contact angles of the catalytic layer before and after electrolysis. Unless otherwise stated, the reactions were conducted under identical conditions.

**Figure S20. Effect of catalyst loading on the wettability and styrene hydrogenation performance of the PME.** (a–c) Schematic illustrations of PMEs with increasing catalyst loading. (d–f) Corresponding water contact angles of the catalytic-layer surfaces. (g–i) Styrene hydrogenation performance of the corresponding PMEs.

**Note:** Increasing the catalyst loading caused only a minor change in the water contact angle (116.14°–112.42°), whereas the styrene hydrogenation performance became more stable. These results suggest that the improved reaction stability is not primarily governed by macroscopic surface wettability, but is associated with the increased catalyst loading and corresponding catalytic-layer thickness.

**Figure S21.** Electricity consumption as a function of CE of p-aminophenol and cell voltage.

**Figure S22.** The TEA model used for investigating the production of PAP.

**Figure S23. Sensitivity analysis.** The impact of variations of (a) current density and (b) CE on the capital cost and operating cost. (c) Profit-sensitive analysis of relevant process parameters for PAP electrosynthesis under standard conditions (50 mA cm^−2^, 0.05 US$ kWh^−1^, 2.6 US$ kg^−1^, and 90% CE). (d) The cumulative NPV as a function of year. The NPV = 0 is the breakeven line, and the intersection with the curve represents the payback point.

**Figure S24. LCA models for conventional catalytic hydrogenation, electrochemical synthesis and PME electrosynthesis.**

**Figure S25.** The CO_2_ emission (unit: kg CO_2_e kg^−1^) as a function of CE of PAP and cell voltage via PME reactor when using different energy input sources. (a) Fossil energy, (b) PV, and (c) wind energy. (d) CO_2_ emissions comparison of three different routes (method 1: catalytic hydrogenation, method 2: conventional electrolysis, and method 3: PME electrolysis) employing different types of energy sources. **TEA and LCA for PAP synthesis**

**Supplementary Note 1: Technical indicators**

To provide a preliminary techno-economic benchmark for the PME reactor, quantitative inputs in this TEA were primarily drawn from reported electrochemical syntheses of p-aminophenol across different scales, which provide reliable benchmarks (Table S5) [70, 71].

**Supplementary Note 2: Calculation of electricity consumption of electrosynthesis process**

The electricity consumption (E, MJ kg^−1^) is calculated based on the equation:

$E=Q\times U$	(1)

where Q is the charge required to produce 1 kg of PAP and U is the cell voltage.

The charge required can be calculated as follows:

$Q=\frac{mole of PAP\times F\times Ne}{CE}$	(2)

The energy consumed can be calculated as follows:

$$E=Q\times U=\frac{\frac{1000}{109.13} mol\times96485 C{mol}^{-1}\times4}{CE}\times U$$

$=3.54\times{10}^{6}\times\frac{U}{CE} J per kg of PAP$	(3)

Therefore, a generalized relation between the electricity consumption, (Z, unit: MJ kg^−1^ PAP), and two arguments, i.e., the CE of PAP (X) and the electrolyzer voltage (Y, unit: V), can be described as (Fig. S21):

$Z=3.54Y/X$	(4)

**Supplementary Note 3: Techno-economic analysis (TEA) of the electrosynthesis process**

In parallel, we have referred to the established TEA studies in related fields as methodological templates, ensuring consistency and comparability [72–79]. These references form the basis for the present analysis and support a transparent, assumption-based assessment of the potential economic and environmental performance of the PME process. A scenario-based set of assumptions were adopted for the production of 1 t of PAP, with the purpose of enabling a first-order comparison among routes.

*Assumptions*:

1. Unless otherwise stated, the base-case TEA assumes a representative operating point of 90% current efficiency; 3.23 V cell voltage, and 50 mA cm^−2^ current density derived from the present experimental dataset (Fig. 3h); these values are used for scenario comparison rather than to represent the full operating envelope.
2. The lifetime of the plant is assumed to be 10 years, and the working time is set as 350 days per year. The cost of balance of plant is assumed to be 50% of electrolyzer cost [75].
3. The separation equipment cost is set as 30% of the electrolyzer cost (Table. S7).
4. The electricity price is assumed to be 0.05 $ kWh^−1^ [80].
5. The maintenance cost is set as 2.5% of the capital cost [81].
6. The market price of PAP was treated as a scenario variable, with set a base value of 2.6 $ kg^−1^ in the sensitivity analysis.

To produce 1 ton of PAP, some basic parameters can be calculated.

We first calculated the total charge required. The CE of PAP is 90% (3.23 V, 50 mA cm^−2^) obtained from PME reactor.

$$Q=\frac{Mass of PAP\times F\times n}{Molar mass of PAP\times CE}$$

$=\frac{1\times{10}^{6}g\times96485 C{mol}^{-1}\times4}{109.13 g{mol}^{-1}\times90\%}=3.9\times{10}^{9}C$	(4)

Then, the total current required was calculated as follow:

$I=\frac{Q}{t}=\frac{3.9\times{10}^{9}C}{24h\times3500}=46781 A$	(5)

We can calculate the area of electrolyzer required based on eqn. (5).

$Electrolyzer area=\frac{I}{current density}=\frac{46781 A}{0.05 A{cm}^{-2}}=93.56m^{2}$	(6)

The power required is calculated as follows:

$Power=3.23 V\times46781 A=151.10 kW$	(7)

**Supplementary Note 4: Total cost to produce 1t PAP**

The total cost was partitioned into four components: capital cost, operating cost, materials cost, and cost adjustments (Fig. S22) [77, 80].

1. **Materials cost:**

Comparisons with catalytic hydrogenation and conventional electrolysis are provided in Table S6.

1) Nitrobenzene

To produce 1 ton of p-aminophenol per day, 1.1 ton of nitrobenzene is consumed.

$Cost of nitrobenzene=1.1 ton of nitrobenzene\times983.15 \$ {ton}^{-1}=1091.29 \$ {ton}^{-1} {day}^{-1}$	(9)

2) H_2_SO_4_

To produce 1 ton of p-aminophenol per day, 2.4 ton of H_2_SO_4_ is consumed.

$Cost of 20\% sulfuric acid solution=2.4 ton of \mathrm{sulfuric}\mathrm{acid}\times49.44 \$ {ton}^{-1}=118.66 \$ {ton}^{-1} {day}^{-1}$	(10)

3) Ammonium water

To produce 1 ton of p-aminophenol per day, 1.1 ton of ammonium water is consumed.

$$Cost of ammonium water=0.8 ton of nitrobenzene\times140.45 \$ {ton}^{-1}$$

$=112.36 \$ {ton}^{-1}{day}^{-1}$	(11)

4) Active carbon

To produce 1 ton of p-aminophenol per day, 0.09 ton of active carbon is needed.

$$Cost of active carbon=0.09 ton of nitrobenzene\times1123.60 \$ {ton}^{-1}$$

$=101.12 \$ {ton}^{-1} {day}^{-1}$	(12)

$$Materials cost of PAP per ton=1091.29+118.66+112.36+101.12$$

$=1423.43 \$ {ton}^{-1}{day}^{-1}$	(13)

2. **Capital cost:**

*The capital recovery factor (CRF) was calculated as:*

$CRF=\frac{r{（1+r）}^{n}}{{（1+r）}^{n}-1}=\frac{10\%{\times（1+10\%）}^{10}}{{（1+10\%）}^{10}-1}=0.1627$	(14)

where *r* (discount rate*)* = 10%, n (equipment lifetime) = 10.

1) Electrolyzer

The electrolyzer cost was estimated based on the area-specific capital cost ($ m⁻²) following the methodology reported [82, 83].The total cost was obtained by multiplying the unit cost per active area with the total electrode area and applying a scale-up factor (fₛ = 1.12) and capital recovery factor (CRF).

The electrolyzer capital cost was determined based on the component-level area-specific costs including the pressure plate, gasket, porous membrane electrode (PME), counter electrode (DSA), ion-exchange membrane, collector plate, frame, and sealing parts (TEA spreadsheet for details) [83].

$Electrolyzer cost per area=2883.42 \$ m^{-2}\times1.12=3215.02 \$ m^{-2}$	(15)

The electrolyzer was calculated below for reference:

$Electrolyzer cost=93.56m^{2}\times3215.02 \$ m^{-2}=300803.88 \$$	(16)

2) Separation equipment

In catalytic hydrogenation and conventional electrolysis, separation units (e.g., multiphase separation, catalyst recovery, distillation) contribute significantly to capital cost because of the need to separate organic/aqueous phases, recycle catalysts, and purify intermediates. Capital costs were allocated accordingly, with separation equipment assumed to represent 60% of the installed reactor cost for catalytic hydrogenation and 50% for conventional electrolysis (Table S8).

By contrast, PME electrolysis is expected to simplify several downstream operations related to, electrolyte handling and phase separation, and is therefore modeled here using a reduced separation train consisting of neutralization, distillation, crystallization and drying step. Therefore, the separation equipment was conservatively assumed to be 30% of the electrolyzer cost.

$Separation Equipment =300803.88 \$\times30\%=90241.16 \$$	(17)

3) Balance of plant (BOP)

Balance-of-plant (BOP) costs cover essential components like the electrical supply system, pumps, storage, piping, heat management, safety systems, and structural elements. These are necessary for stable operation but exclude separation units, which are separately accounted for.

$Cost of balance of plant =300803.88 \$\times50\%=150401.94 \$$	(18)

$$Capital cost=\frac{\left( {cost}_{electrolyzer}+{cost}_{separation equipment}+{cost}_{balance of plant} \right)\times CRF}{m_{PAP} per day}$$

$=\frac{(300803.88 \$+90241.16 \$+150401.94 \$)\times0.1627}{350 day\times1 ton}=251.77 \$ {ton}^{-1} {day}^{-1}$	(19)

3. **Operating cost:**
1. Electricity

$Electricity cost=151.1 kW\times24 h\times0.05 \$ {kWh}^{-1}=181.3 \$ {ton}^{-1}{day}^{-1}$

(20)

2. Maintenance

$$Maintenance cost=286.18 \$ {ton}^{-1} {day}^{-1}\times2.5\%$$

$=6.29 \$ {ton}^{-1} {day}^{-1}$	(21)

3. Utilities

To benchmark the three routes on an equal basis, utilities were allocated following typical industrial practice. Electrical energy covers DC power to the electrolyzer, rectifier losses, and all motor loads (compressors and pumps). Thermal duties for separations—including rectification, solvent recovery, evaporation, and drying—were assigned to steam, with no steam-driven pumps considered. Rectification was treated as the dominant thermal duty and allocated to steam, with a benchmark value of 2049 kWh t⁻¹ adopted for both conventional and PME electrosynthesis.

For PME, this is treated as a conservative modeling assumption reflecting a simplified scenario with reduced biphasic extraction and solvent/catalyst recovery. For downstream separation, the benchmark was conventional electrolysis (534 kWh t⁻¹, Table S5). PME electrolysis was conservatively assumed to require 65% of this value, reflecting its simplified flowsheet without biphasic extraction, solvent or catalyst recovery, and with minimal wastewater generation. The theoretical electrolyzer demand estimated from E = QU at 3.23 V and 90% CE corresponds to ~18–20 MJ kg^−^¹, consistent with the site-level electricity of ~27 MJ kg⁻^1^ in Table S5 once rectifier losses and auxiliary loads are included.

$$Operating cost=Electricity cost+Maintenance cost+Utilities cost$$

$=181.30+6.29+98.56=286.18 \$ {ton}^{-1}{day}^{-1}$	(22)

4. Cost adjustments

Cost adjustments were introduced as route-specific overhead factors applied to direct operating expenditures (DOE, defined as the sum of raw materials and utilities costs):

$$Cost adjustment=\beta\times DOE$$

Overhead adjustments were then applied as proportional factors β to DOE, consolidating safety, compliance, wastewater treatment, and, where applicable, catalyst and solvent recovery.

Catalytic hydrogenation carries an additional burden from high-pressure hydrogen management and noble-metal recovery ($\beta_{cat.}=0.15$); conventional electrolysis carries the highest overhead, dominated by downstream separation and solvent recycling ($\beta_{con.}=0.20$); while PME electrolysis is assigned the lowest factor in this model, reflecting an assumed simplified flowsheet without solvent or catalyst recovery and minimal wastewater generation ($\beta_{PME}=0.10$).

These coefficients are assumed values chosen to reflect relative process complexity and to capture the essential economic distinctions among the three routes.

$$DOE=materials cost+utilities cost$$

$=1423.43+98.56=1521.99 \$ {ton}^{-1}{day}^{-1}$	(23)

$Cost adjustment=1521.99 \$ {ton}^{-1} {day}^{-1}\times0.1=152.20 \$ {ton}^{-1} {day}^{-1}$ (24)

**The total cost** = Materials cost + Capital cost + Operating cost + Cost adjustments = 1423.43 + 251.77 + 286.18 + 152.20= 2113.58 $ ton⁻^1^ day⁻^1^

The daily profit is calculated below:

**Daily profit** = income – cost = 2600 $ ton⁻^1^ day⁻^1^ – 2113.58 $ ton⁻^1^ day⁻^1^

= 486.42 $ ton⁻^1^ day⁻^1^

The total cost analysis of the three technology routes (catalytic hydrogenation, conventional electrolysis, and PME electrolysis) in Table S8 was derived from multiple sources (TEA spreadsheet for calculation details). Feedstock prices were taken from Table S3. The balance of plant (BOP) was uniformly set at 50% across all routes. Remaining cost elements, including electricity, maintenance, and utilities, were estimated following the PME framework to ensure consistency in methodology.

**Supplementary Note 5: Sensitivity analysis**

Four sensitive factors (current density, CE, electricity price, and market price of PAP) are evaluated in sensitivity analysis. The base values of these four factors are set as 50 mA cm^−2^, 90%, $0.05 kWh^−1^, and $3.5 kg^−1^, respectively. The fluctuation range is from −75% to 100%. The cell voltage is fixed at 3.23 V. While investigating the impact of one parameter, the rest of the parameters were kept at the base value (Fig. S22a–c).

**Supplementary Note 6: NPV method for evaluating the payback time of initial capitals**

The end-of life NPV can be calculated using the following formula [80, 81]:

$$NPV=\sum_{i=0}^{n} \frac{CF_{i}}{{(1+r)}^{i}}$$

where CF_i_ is the cash flow in the year i, r is the discount rate (r = 10%), and n is the plant lifetime (n = 10 in this work). If NPV < 0, a project will have a negative profitability potential, whereas NPV > 0 indicates the project is economically viable (Fig. S14d).

The CF_i_ is calculated from the following equation:

$$CF_{i}=P_{i}-Tax_{i}$$

where $P_{i}$ is the yearly profit, which equals to the difference between income and cost:

$$P_{i}={Income}_{i}-{Cost}_{i}$$

While $Tax_{i}$ can be calculated as follows:

$${Tax_{i}}_{i}=(CF_{i}-{Dep}_{i})\times t$$

where ${Dep}_{i}$ is the depreciation of the initial capitals (10% salvage value and a linear depreciation are employed); $t$ is the tax rate (39.8%).

**Supplementary Note 7: Carbon Emission Life-cycle Assessment (LCA)**

This analysis is intended as a scenario-based carbon-footprint comparison rather than a comprehensive multi-indicator life-cycle sustainability assessment. The LCA model contains cradle-to-grave CO_2_ emissions of PAP mainly from three aspects: raw materials, production process, and end-use. For fair comparison, we harmonized system boundaries across routes: upstream H_2_ production + compression (for catalytic hydrogenation), precious-metal catalyst manufacture/recycling, cosolvent production & recovery, and wastewater treatment are included. For PME, the avoided downstream steps (no cosolvent/catalyst recovery, minimal liquid–liquid extraction) are credited as reduced steam and utility demand.

Fig. S23 shows the LCA model of conventional catalytic hydrogenation process and electrochemical process for PAP, respectively [58, 85]. To start, the related data were collected. Table S10 shows the carbon emission intensities of the feedstocks and the energy sources, etc., which are obtained from Ecoinvent 3.9.1 database. The carbon emission factor of the grid (0.581 kg CO_2_e kWh⁻^1^) in China is obtained from the Chinese government's official file.

1. Inventory of conventional catalytic hydrogenation process

1) In the conventional catalytic hydrogenation process, the CO_2_ emissions from raw materials mainly contain that from the production of nitrobenzene, noble metal (Pt/C), sulfuric acid, cosolvent, hydrogen gas (H_2_), and ammonium water. The carbon intensities of these feedstocks are listed in Table S10.
2) The CO_2_ emissions from the production process mainly come from fossil energy consumption. We assume that the energy supply is derived from the grid that is from coal burning. And the CO_2_ emission of coal mining is also considered [81]. The CO_2_ emission factor of the grid in China is 0.581 kg CO_2_e kWh⁻^1^. The CO_2_ emission from coal mining per kWh of electricity is 0.0367 kg CO_2_e kWh⁻^1^.
3) Additional contributions arise from the use and recycling of noble-metal catalysts, as well as the handling of high-pressure hydrogen (2–5 MPa). These factors not only increase the indirect emissions but also introduce safety risks during large-scale operation.

The total CO_2_ emissions inventory of the catalytic hydrogenation process is presented in Table S11.

2. Inventory of conventional electrolysis process

1) In the conventional electrolysis process, the CO_2_ emissions from raw materials mainly contain that from the production of nitrobenzene, emulsifier (DDAO), sulfuric acid, ammonium water and active carbon. The carbon intensities of these feedstocks are listed in Table S10.
2) The dominant source of CO_2_ emissions during the process operation is electricity consumption. Similar to the catalytic hydrogenation route, the grid emission factor (0.581 kg CO_2_e kWh⁻^1^) and coal mining emissions (0.0367 kg CO_2_e kWh⁻^1^) are applied.
3) Compared with catalytic hydrogenation, conventional electrolysis avoids the use of compressed hydrogen and noble-metal catalysts, but it suffers from significantly higher electricity demand. This not only results in elevated carbon intensity but also creates additional downstream separation challenges that indirectly contribute to the environmental footprint.

The total CO_2_ emissions inventory of the conventional electrolysis process is presented in Table S12.

3. Inventory of PME electrolysis process

1) In the PME electrolysis process, the CO_2_ emissions from raw materials mainly contain that from the production of nitrobenzene, sulfuric acid, ammonium water and active carbon. The carbon intensities of these feedstocks are listed in Table S10.
2) The dominant source of CO_2_ emissions during the process operation is electricity consumption. Besides the grid emission factor (0.581 kg CO_2_e kWh⁻^1^), coal mining emissions (0.0367 kg CO_2_e kWh⁻^1^) are applied. Wind, and photovoltaic electricity are also considered. The CO_2_ emissions generated from the construction of the electrolyzer or the separation equipment are negligible, since these are far lower than the emissions from the materials and operation.
3) Compared with catalytic hydrogenation and conventional electrolysis, PME electrolysis not only avoids the use of compressed hydrogen and noble-metal catalysts, but also alleviates downstream separation burden that indirectly contributes to the environmental footprint.

The total CO_2_ emissions inventory of the PME electrolysis process is presented in Table S13.

**Supplementary Note 8: Additional quantitative sustainability metrics**

To complement the techno-economic analysis and carbon-footprint assessment, additional quantitative sustainability metrics were introduced to further compare catalytic hydrogenation, conventional electrolysis, and PME electrolysis for p-aminophenol (PAP) production. A common functional unit of 1 kg PAP was adopted for all calculations. The additional metrics were selected to provide complementary information on material efficiency, auxiliary-material demand, downstream separation burden, and representative environmental and fossil-resource impacts.

The analysis was based primarily on the process material and energy inventories summarized in Tables S6 and S8. For the screening life-cycle impact assessment, cumulative characterization factors from the ecoinvent 3.9.1 database under the CML v4.8 2016 framework were used. China-specific datasets were selected where available; otherwise, Rest-of-World (RoW) or global datasets were used consistently for all three production routes.

1. Gross process material intensity

Process mass intensity (PMI) is commonly defined as the total mass of materials used per unit mass of product. Because the present process model does not explicitly account for complete recycle streams, water consumption, and all downstream material flows, the metric used here is termed gross process material intensity (GPMI) rather than formal PMI.

The GPMI was calculated as:

$$GPMI=\frac{\sum_{i} m_{i}}{m_{PAP}}$$

where $m_{i}$ is the mass of each material input included in the modeled process inventory and $m_{PAP}$ is the mass of PAP produced.

Based on the inventories in Table S5, the calculated GPMI values were:

${GPMI}_{CH}=1.11+1.80+0.001+0.05+0.02+1.00=3.98$	(25)

${GPMI}_{CE}=1.11+2.40+0.02+0.09+0.8=4.42$	(26)

${GPMI}_{PME}=1.11+2.40+0.09+0.05+0.8=4.40$	(27)

where CH, CE, and PME denote catalytic hydrogenation, conventional electrolysis, and PME electrolysis, respectively.

Although the GPMI of PME electrolysis is similar to that of conventional electrolysis, this metric only reflects the gross mass of process inputs and does not account for differences in material function, environmental burden, or downstream processing requirements.

2. Auxiliary-material intensity

To further distinguish the consumption of non-feedstock process aids, an auxiliary-material intensity (AMI) was defined as:

$$AMI=\frac{\sum_{i} m_{aux,i}}{m_{PAP}}$$

where $m_{aux,i}$ represents the mass of catalysts, adsorbents, cosolvents, and emulsifiers used in the process.

Nitrobenzene, H2SO4, H2, and aqueous ammonia were treated as principal reactants or process reagents and were therefore excluded from the AMI calculation.

For catalytic hydrogenation, the auxiliary-material input includes Pt/C catalyst and cosolvent:

$${AMI}_{CH}=0.001+0.020=0.021 \mathrm{kg}\mathrm{kg}^{-1}\mathrm{PAP}$$

For conventional electrolysis, the auxiliary-material input includes cosolvent/emulsifier and activated carbon:

$${AMI}_{CE}=0.02+0.090=0.110 \mathrm{kg}\mathrm{kg}^{-1}\mathrm{PAP}$$

For PME electrolysis, activated carbon is the principal auxiliary material:

$${AMI}_{PME}=0.09 \mathrm{kg}\mathrm{kg}^{-1}\mathrm{PAP}$$

The relative reduction in AMI for PME electrolysis compared with conventional electrolysis was calculated as:

$$R_{AMI}=\frac{{AMI}_{CE}-{AMI}_{PME}}{{AMI}_{PME}}\times100\%=18.2\%$$

Thus, under the modeled scenario, PME electrolysis reduces auxiliary-material demand by approximately 18.2% compared with conventional electrolysis.

3. Separation-energy intensity

Separation-energy intensity (SEI) was defined as the downstream separation-energy demand per unit mass of PAP produced. The corresponding values were 652.67, 534.00, and 347.10 kWh t⁻¹ PAP for catalytic hydrogenation, conventional electrolysis, and PME electrolysis, respectively (Table S8), corresponding to reductions of 46.8% and 35.0% for PME electrolysis relative to catalytic hydrogenation and conventional electrolysis, respectively. These values indicate that the modeled PME route requires substantially less downstream separation energy than the two comparison routes, consistent with its simplified phase-management and recovery requirements.

4. Screening life-cycle impact assessment

To extend the environmental comparison beyond carbon emissions, two representative midpoint indicators—acidification potential (AP) and abiotic depletion of fossil fuels (ADP-fossil)—were evaluated using cumulative CML v4.8 2016 characterization factors from the ecoinvent 3.9.1 database. Following the same functional unit of 1 kg PAP, each impact was calculated as the sum of the process input multiplied by its corresponding characterization factor:

$$I_{j}=\sum_{i} A_{i}\times CF_{i,j}$$

where $A_{i}$ is the consumption of material or energy input ii per kilogram of PAP and $CF_{i,j}$ is the cumulative characterization factor.

For acidification potential, the three routes were calculated as:

$${AP}_{CH}=\left( 1.11\times0.01863 \right)+\left( 1.80\times0.01041 \right)+\left( 0.001\times205.90 \right)$$

$+\left( 0.05\times0.01296 \right)+\left( 0.02\times0.00942 \right)+\left( 1.00\times0.01633 \right)$

$+\left( 0.1497\times0.00413 \right)=0.2631 \mathrm{kg}{\mathrm{SO}_{2^{-}}eq kg}^{-1}\mathrm{PAP}$	(28)

$${AP}_{CE}=\left( 1.11\times0.01863 \right)+\left( 2.40\times0.01041 \right)+\left( 0.090\times0.01965 \right)$$

$+\left( 0.80\times0.01633 \right)+\left( 7.056\times0.00413 \right)$

$=0.08967 \mathrm{kg}{\mathrm{SO}_{2^{-}}eq kg}^{-1}\mathrm{PAP}$	(29)

$${AP}_{PME}=\left( 1.11\times0.01863 \right)+\left( 2.40\times0.01041 \right)+\left( 0.090\times0.01965 \right)$$

$+(0.80\times0.01633)+(4.711\times0.00413)$

$=0.07998 \mathrm{kg}{\mathrm{SO}_{2^{-}}eq kg}^{-1}\mathrm{PAP}$	(30)

For fossil-resource depletion:

$${ADP}_{fossil,CH}=\left( 1.11\times54.87 \right)+\left( 1.80\times2.09 \right)+\left( 0.001\times49584.1 \right)$$

$+\left( 0.050\times145.03 \right)+\left( 0.020\times60.19 \right)+\left( 1.00\times40.67 \right)$

$+\left( 0.1497\times8.49 \right)=164.64 MJ\mathrm{kg}^{-1}\mathrm{PAP}$	(31)

$${ADP}_{fossil,CE}=(1.11\times54.87)+(2.40\times2.09)+(0.090\times37.11)$$

$+\left( 0.80\times40.67 \right)+\left( 7.056\times8.49 \right)=161.69 MJ\mathrm{kg}^{-1}\mathrm{PAP}$	(32)

$${ADP}_{fossil,PME}=(1.11\times54.87)+(2.40\times2.09)+(0.090\times37.11)$$

$+(0.80\times40.67)+(4.711\times8.49)=141.78 MJ\mathrm{kg}^{-1}\mathrm{PAP}$	(33)

Thus, PME electrolysis shows reductions of approximately 69.6% and 10.8% relative to catalytic hydrogenation and conventional electrolysis, respectively. PME electrolysis reduces the modeled fossil-resource depletion by approximately 13.9% relative to catalytic hydrogenation and 12.3% relative to conventional electrolysis.

The Pt/C characterization factor was estimated from 5 wt.% Pt and 95 wt.% activated carbon. For conventional electrolysis, the contribution of DDAO was not included because an exact corresponding dataset was not identified in the ecoinvent database. These results are intended as a screening-level LCIA comparison rather than a comprehensive multi-indicator LCA.

**Table S1.** Effect of operating parameters on the styrene hydrogenation performance.

| Entry | Liquid level difference | Current density (mA cm^−2^) | Temperature | Oil phase composition | Current efficiency |
| --- | --- | --- | --- | --- | --- |
| 1 | −1 cm | 30 | 50 ℃ | Pure styrene | N.D. |
| 2 | 0 cm | 30 | 50 ℃ | Pure styrene | 95.2% |
| 3 | 1 cm | 30 | 50 ℃ | Pure styrene | 99.0% |
| 4 | 10 cm | 30 | 50 ℃ | Pure styrene | 85.7% |
| 5 | 38 cm | 30 | 50 ℃ | Pure styrene | 90.4% |
| 6 | 1 cm | 100 | 50 ℃ | Pure styrene | 91.4% |
| 7 | 1 cm | 50 | 50 ℃ | Pure styrene | 87.9% |
| 8 | 1 cm | 30 | 5 ℃ | Pure styrene | 82.5% |
| 9 | 1 cm | 30 | 25 ℃ | Pure styrene | 76.5% |
| 10 | 1 cm | 30 | 90 ℃ | Pure styrene | 88.5% |
| 11 | 1 cm | 30 | 50 ℃ | 0.1 wt.% ST | 53.1% |
| 12 | 1 cm | 30 | 50 ℃ | 1 wt.% ST | 56.6% |
| 13 | 1 cm | 30 | 50 ℃ | 20 wt.% ST | 65.8% |
| 14 | 1 cm | 30 | 50 ℃ | 60 wt.% ST | 76.4% |
| 15 | 1 cm | 30 | 50 ℃ | 20 wt.% ST  in MIBK | 76.1% |
| 16 | 1 cm | 30 | 50 ℃ | 20 wt.% ST  in BA | 93.2% |
| 17 | 1 cm | 30 | 50 ℃ | 20 wt.% ST  in DCM | 87.2% |
| 18^a^ | 1 cm | 30 | 50 ℃ | Pure styrene | 49.6% |
| 19^b^ | 1 cm | 30 | 50 ℃ | Pure styrene | 61.6% |

Unless otherwise stated, 10 wt.% H_2_SO_4_ aqueous solution was used as aqueous phase.

^a^10 wt.% NaCl aqueous solution was used as aqueous phase. ^b^1 M NaOH aqueous solution was used as aqueous phase.
**Table S2.** Porous membrane electrodes used for representative electro-organic transformations.

| Reaction | PME electrode  (catalyst loading) | Electrode area (cm^2^) | Ink amount per coating area  (mL cm⁻²) |
| --- | --- | --- | --- |
| Reduction reaction | | | |
|  | hbCP/Pt-CNT  (0.8 mg cm^−2^) | 60 | 0.83 |
|  | hbCP/Pd-C  (1.0 mg cm^−2^) | 3.14 | 10.0 |
|  | hbCP/Pd-C  (0.5 mg cm^−2^) | 60 | 0.83 |
|  | hbCP/Cu  (1 mg cm^−2^) | 3.14 | 10.0 |
|  | hbCP/Pt-C  (1 mg cm^−2^) | 3.14 | 10.0 |
|  | hbCP/Pt-C  (0.5 mg cm^−2^) | 60 | 0.83 |
|  | hbCP/Cu  (1 mg cm^−2^) | 60 | 0.83 |
|  | hbCP/Cu-S  (0.5 mg cm^−2^) | 60 | 0.83 |
|  | hbCP/Pd-C  (0.5 mg cm^−2^) | 60 | 0.83 |
|  | hbCP/Ag  (0.5 mg cm^−2^) | 60 | 0.83 |
|  | hbCP/Ag  (0.5 mg cm^−2^) | 60 | 0.83 |
|  | hbCP/Cu  (1.0 mg cm^−2^) | 3.14 | 10.0 |
| Oxidation reaction | | | |
|  | hbCP/Pt-C  (0.5 mg cm^−2^) | 3.14 | 10.0 |
|  | hbCP/Pt-C  (0.5 mg cm^−2^) | 3.14 | 10.0 |
|  | hbCP/Pt-C  (0.5 mg cm^−2^) | 60 | 0.83 |
|  | hbCP | 60 | 0.83 |
|  | hbCP | 60 | 0.83 |
|  | hbCP/β-Ni(OH)_2_  (2.0 mg cm^−2^) | 3.14 | 10.0 |
|  | hbCP/β-Ni(OH)_2_  (2.0 mg cm^−2^) | 60 | 0.83 |
|  | hbCP/β-Ni(OH)_2_  (2.0 mg cm^−2^) | 60 | 0.83 |
|  | hbCP | 60 | 0.83 |

Unless otherwise specified, YLS-30T carbon paper was used as the porous conductive substrate. For PMEs with effective areas of 3.14 and 60 cm^2^, the coating areas (ink volumes) were 4 cm^2^ (40 mL) and 60 cm^2^ (50 mL), corresponding to ink amounts of 10.0 and 0.83 mL cm^−2^, respectively. Catalyst loading refers to the deposited catalyst mass per effective electrode area.

**Table S3.** Phase crossover and physicochemical evolution during extended PME operation.

| Phase/property | Parameter | Initial | After |
| --- | --- | --- | --- |
| Organic phase | Water content (wt.%) | 0.02 | 0.08 |
|  | Sulfate crossover | N.D. | N.D. |
| Aqueous electrolyte | Styrene crossover | N.D. | N.D. |
|  | Ethylbenzene crossover | N.D. | N.D. |
|  | Leached Pt concentration (mg L^−1^) | - | 1.37 ± 0.07 |
|  | pH | 0.13 | −0.09 |
|  | Conductivity (ms cm^−1^) | 39.4 | 51.4 |
| PME surface | Contact angle | 144.53° | 114.57° |

Note: Water content was determined by Karl Fischer titration, sulfate by ion chromatography, styrene and ethylbenzene by GC, and Pt by ICP-OES. N.D. indicates not detected under the corresponding analytical conditions. The Pt concentration after operation corresponds to approximately 5.3% of the initial Pt inventory, calculated using a 100 mL electrolyte volume and an initial Pt loading of 2.60 mg.

**Table S4.** Performance and product analysis of the 455 cm^2^ PAP scale-up experiment.

| Parameter |  |
| --- | --- |
| Reactor configuration | Multi-module plate-frame PME reactor |
| Total electrode area | 455 cm^2^ (5 × 91cm^2^) |
| Reaction temperature | 80 °C |
| Current density | 50 mA cm^−2^ |
| Total current | 22.75 A |
| Reaction time | 30 h |
| Total charge passed | 682.5 Ah (2.457 × 10^6^ C) |
| Organic phase | Pure nitrobenzene |
| Aqueous electrolyte | 20 wt.% H_2_SO_4_ |
| Current efficiency of PAP | 83.5% |
| PAP selectivity | 84.1% |
| Purified PAP mass | 447.4 g |
| PAP purity | 98.9% (HPLC) |
| Current efficiency of AN | 16.4% |
| Azobenzene | N.D. (not detected) |
| Azoxybenzene | N.D. (not detected) |
| Aqueous-phase nitrobenzene | 68.18 mM |
| Quantified product CE balance | 99.9% |

N.D., not detected under the analytical conditions used. The aqueous-phase nitrobenzene concentration was measured after electrolysis and does not represent the total residual substrate. Because neat nitrobenzene was used in excess as both the reactant and organic phase, a conventional substrate-based conversion or isolated yield was not calculated.

**Table S5.** Technical indicators comparison of different synthesis routes.

|  | Catalytic  Hydrogenation | Conventional electrosynthesis | PME electrolysis  (This work) |
| --- | --- | --- | --- |
| Temperature | 90~120 ℃ | 90 ℃ | 80 ℃ |
| Pressure | 2~5 Mpa (H_2_) | 0.1 Mpa | 0.1 Mpa |
| Yield | 85–90% | 85–90% | 85–90% |
| CE | / | 85–90% | ~90% |
| Product purity | 95–97% | 96–98.5% | 98% |
| Average cell voltage | / | 4.45 V | 3.23 V |

**Table S6.** Raw materials for 1 t PAP.

| Raw materials | Unit Price  ($·t^−1^) | Catalytic hydrogenation (t/$) | Conventional electrosynthesis  (t/$) | PME electrolysis  (This work)  (t/$) |
| --- | --- | --- | --- | --- |
| NB | 983.15 | 1.11/1,091.29 | 1.11/1,091.29 | 1.11/1,091.29 |
| H_2_SO_4_ | 49.16 | 1.80/88.99 | 2.40/118.66 | 2.40/118.66 |
| Noble metal（Pt/C） | 22,191.01 | 0.0010/18.82* | / | / |
| H_2_ | 2,808.99 | 0.050/140.45 | / | / |
| Cosolvent | 3,511.24 | 0.020/70.22 | 0.02/70.22 | / |
| Activated carbon | 1,123.60 | / | 0.09/101.12 | 0.090/101.12 |
| Liquid ammonia | 140.45 | 1.00/140.45 | 0.8/112.36 | 0.8/112.36 |
| Total |  | 1,516.58 $ | 1,493.66 $ | 1,423.43 $ |

**Table S7.** Detailed equipment breakdown.

| Items | Catalytic hydrogenation | Conventional electrolysis | PME electrolysis |
| --- | --- | --- | --- |
| Extraction and solid–liquid separation unit | √ | √ | Х |
| Catalyst recovery | √ | Х | Х |
| Solvent recovery | √ | √ | Х |
| Neutralization | √ | √ | √ |
| Salt recovery | √ | √ | √ |
| Drying | √ | √ | √ |

**Table S8.** Energy consumption and cost for production of 1 t PAP.

|  | Item | Price  ($·kWh^−1^) | Catalytic hydrogenation  (kWh t^−1^/$) | Conventional electrosynthesis  (kWh t^−1^/$) | PME electrolysis  (kWh t^−1^/$) |
| --- | --- | --- | --- | --- | --- |
| Electric energy | Electrolysis | 0.050 | / | 4958.43/247.92 | 3599.04/179.95 |
|  | Line loss |  | / | 962.00/48.10 | 962.00/48.10 |
|  | Cathode rotation |  | / | 116.00/50.80 | / |
|  | H_2_ compressor |  | 121.2/6.06 | / | / |
|  | Pumps |  | 28.5/1.43 | 120.00/6.00 | 150.00/7.50 |
|  | Total |  | 149.7/7.48 | 7056.43/352.82 | 4711.04/235.55 |
| Chemical energy | H_2_ | 0.084 | 1,665/139.86 | / | / |
| Heat energy | Rectification | 0.035 | / | 2049.00/71.72 | 2049.00/71.72 |
|  | Separation |  | 652.67/22.84 | 534.00/18.69 | 347.10/12.15 |
|  | Drying |  | 300.00/10.50 | 300.00/10.50 | 300.00/10.50 |
|  | Other  (heating) |  | 500/17.50 | 150/5.25 | 120/4.20 |
| Total |  |  | 3267.37/198.19 | 10089.43/458.98 | 7527.04/334.11 |

Notes: we postulated unit prices: H₂ chemical energy = 0.084 $·kWh^−1^; steam = 0.035 $·kWh^−1^.

Hydrogen chemical energy was calculated from the lower heating value (LHV) of hydrogen:

$E_{H2}={LHV}_{H2}\times m_{H2}= 33.3 t^{-1}\times0.05 t H₂ per ton PAP=1,665 kWh$ $t^{-1}$

Heat energy (steam) was estimated based on typical steam consumption in fine chemical production:

$$E_{heat}=m_{steam}\times h_{steam}=1.0 t per ton PAP\times500 kWh t^{-1}$$

$=500 kWh t^{-1}$

**Table S9.** Breakdown of costs for catalytic hydrogenation, conventional electrolysis, and PME electrolysis.

| Components | Items | Catalytic hydrogenation  ($ t⁻^1^ day⁻^1^) | Conventional electrolysis  ($ t⁻^1^ day⁻^1^) | PME electrolysis  ($ t⁻^1^ day⁻^1^) |
| --- | --- | --- | --- | --- |
| Materials costs |  | 1,516.58 | 1,493.66 | 1,423.43 |
| Capital costs | Electrolyzer  or stir tank | 23.24 [a] | 46.50 | 139.83 |
|  | Separation equipment | 226.90 | 23.25 | 41.95 |
|  | Balance of plant | 11.62 | 83.70 | 69.92 |
| Operating costs | Electricity cost | 179.64 | 266.65 | 181.32 |
|  | Maintenance | 6.54 | 2.09 | 6.29 |
|  | Utilities | 190.70 | 106.16 | 98.56 |
| Cost adjustment |  | 256.09 | 319.96 | 152.20 |
| Total cost |  | 2,411.32 | 2,272.22 | 2,113.50 |

^a^ In the absence of directly applicable literature data, the equipment cost was treated as a scenario-based assumption for comparative analysis.

**Table S10.** Equivalent carbon emission constants for LCA.

| Items | Values | Unit |
| --- | --- | --- |
| Nitrobenzene | 2.08 | kg CO_2_e kg^−1^ |
| H_2_SO_4_ | 0.14 | kg CO_2_e kg⁻^1^ |
| Noble metal (Pt/C) | 3476.97 | kg CO_2_e kg⁻^1^ |
| 2-Propanol | 3.33 | kg CO_2_e kg⁻^1^ |
| DDAO | 2.74 | kg CO_2_e kg⁻^1^ |
| Hydrogen gas (H_2_) | 30.5 | kg CO_2_e kg⁻^1^ |
| NH_3_H_2_O | 1.69 | kg CO_2_e kg⁻^1^ |
| Active carbon | 7.74 | kg CO_2_e kg⁻^1^ |
| Grid (China) | 0.581 | kg CO_2_e kWh⁻^1^ |
| Coal mining | 0.0367 | kg CO_2_e kWh⁻^1^ |
| Renewable electricity (wind) | 0.0056997 | kg CO_2_e kWh⁻^1^ |
| Renewable electricity (photovoltaic) | 0.01583 | kg CO_2_e kWh⁻^1^ |

**Table S11.** The materials and power subdivided CO_2_ emission per kg of PAP production using industrial catalytic hydrogenation process.

| Materials | Materials | Unit material consumed (kg) | Unit carbon emission  (kg CO_2_e kg^−1^) | Total carbon emission  (kg CO_2_e kg^−1^) |
| --- | --- | --- | --- | --- |
|  | NB | 1.11 | 2.08 | 2.309 |
|  | H_2_SO_4_ | 1.5 | 0.14 | 0.21 |
|  | 5% Pt/C | 0.001 | 3476.97 | 3.477 |
|  | H_2_ | 0.05 | 30.5 | 1.525 |
|  | 2-Propanol | 0.02 | 2.50 | 0.067 |
|  | Liquid ammonia | 1.00 | 1.69 | 1.69 |
| Power | Energy source | Unit energy consumed (kWh) | Unit carbon emission  (kg CO_2_e kWh^−1^) | Total carbon emission  (kg CO_2_e kg^−1^) |
|  | Grid | 0.15 | 0.581 | 0.08715  0.005505 |
|  | Coal mining |  | 0.0367 |  |
| Sum |  |  |  | 9.41 |

**Table S12.** The materials and power subdivided CO_2_ emission per kg of PAP production using conventional electrolysis process.

| Materials | Materials | Unit material consumed (kg) | Unit carbon emission  (kg CO_2_e kg^−1^) | Total carbon emission  (kg CO_2_e kg^−1^) |
| --- | --- | --- | --- | --- |
|  | NB | 1.11 | 2.08 | 2.3088 |
|  | H_2_SO_4_ | 2.4 | 0.14 | 0.336 |
|  | DDAO | 0.02 | 2.74 | 0.0548 |
|  | Activated carbon | 0.09 | 7.74 | 0.6966 |
|  | Liquid ammonia | 0.8 | 1.69 | 1.352 |
| Power | Energy source | Unit energy consumed (kWh) | Unit carbon emission  (kg CO_2_e kWh^−1^) | Total carbon emission  (kg CO_2_e kg^−1^) |
|  | Grid& Coal mining | 7.06 | 0.6177 | 4.36 |
|  | Wind |  | 0.0367 | 0.26 |
|  | Photovoltaic |  | 0.01583 | 0.11 |
| Sum | Grid& Coal mining |  |  | 9.11 |
|  | Wind |  |  | 5.01 |
|  | Photovoltaic |  |  | 4.86 |

**Table S13.** The materials and power subdivided CO_2_ emission per kg of PAP production using PME electrolysis process.

| Materials | Materials | Unit material consumed (kg) | Unit carbon emission  (kg CO_2_e kg^−1^) | Total carbon emission  (kg CO_2_e kg^−1^) |
| --- | --- | --- | --- | --- |
|  | NB | 1.11 | 2.08 | 2.3088 |
|  | H_2_SO_4_ | 2.4 | 0.14 | 0.336 |
|  | Activated carbon | 0.09 | 7.74 | 0.6966 |
|  | Liquid ammonia | 0.80 | 1.69 | 1.352 |
| Power | Energy source | Unit energy consumed (kWh) | Unit carbon emission  (kg CO_2_e kWh^−1^) | Total carbon emission  (kg CO_2_e kg^−1^) |
|  | Grid& Coal mining | 4.71 | 0.6177 | 2.909367 |
|  | Wind |  | 0.0367 | 0.172857 |
|  | Photovoltaic |  | 0.01583 | 0.0745593 |
| Sum | Grid& Coal mining |  |  | 7.60 |
|  | Wind |  |  | 4.87 |
|  | Photovoltaic |  |  | 4.77 |

**Table S14.** Additional quantitative sustainability metrics for PAP production.

| Sustainability metric | Catalytic hydrogenation | Conventional electrolysis | PME electrolysis |
| --- | --- | --- | --- |
| Gross process material intensity  (kg kg^−1^ PAP) | 3.98 | 4.42 | 4.40 |
| Auxiliary-material intensity  (kg kg^−1^ PAP) | 0.021 | 0.110 | 0.090 |
| Separation-energy intensity  (kWh t^−1^ PAP) | 652.67 | 534.00 | 347.10 |
| Acidification potential  (kg SO_2_ eq kg^−1^ PAP) | 0.2631 | 0.08967* | 0.07998 |
| Abiotic depletion, fossil fuels  (MJ kg^−1^ PAP) | 164.64 | 161.69* | 141.78 |

Note: GPMI was calculated from the gross material inputs in Table S6. AMI includes catalysts, adsorbents, cosolvents, and emulsifiers. SEI was calculated from the modeled downstream separation-energy demand in Table S8. AP and ADP-fossil were estimated using cumulative CML v4.8 2016 characterization factors from the ecoinvent 3.9.1 database. China-specific datasets were used where available; otherwise, RoW or global datasets were applied consistently. *The conventional-electrolysis LCIA values exclude DDAO because an exact corresponding ecoinvent dataset was not identified.

Overall, PME electrolysis shows comparable gross material input but lower auxiliary-material demand, separation-energy intensity, acidification potential, and fossil-resource depletion than conventional electrolysis, with reductions of 18.2%, 35.0%, 10.8%, and 12.3%, respectively. Relative to catalytic hydrogenation, the corresponding reductions in separation-energy intensity, acidification potential, and fossil-resource depletion are 46.8%, 69.6%, and 13.9%.
